# Computational explanation for bioactivation mechanism of targeted anticancer agents mediated by cytochrome P450s: A case of Erlotinib

**DOI:** 10.1371/journal.pone.0179333

**Published:** 2017-06-19

**Authors:** Chun-Zhi Ai, Yong Liu, Wei Li, De-Meng Chen, Xin-Xing Zhu, Ya-Wei Yan, Du-Chu Chen, Yi-Zhou Jiang

**Affiliations:** 1 Institute for Advanced Study, Shenzhen University, Shenzhen, Guangdong, China; 2 Key Laboratory of Optoelectronic Devices and Systems of Ministry of Education and Guangdong Province, College of Optoelectronic Engineering, Shenzhen University, Shenzhen, Guangdong, China; 3 School of Life Science and Medicine, Dalian University of Technology, Panjin, Liaoning, China; 4 College of Medicine, Yangzhou University, Yangzhou, Jiangsu, China; 5 School of Dentistry, University of California, Los Angeles, California, United States of America; University of South Alabama Mitchell Cancer Institute, UNITED STATES

## Abstract

EGFR inhibitors, even with therapeutics superiorities in anticancer, can cause idiosyncratic pulmonary and hepatic toxicities that are associated with the reactive electrophile bioactivated by Cytochrome P450s (P450s). Until now, neither has the electrophilic intermediate been caught experimentally, nor has the subtle mechanism been declared. Herein, the underlying mechanism of bioactivation mediated by P450s was explored by DFT calculations for a case of EGFR inhibitor, Erlotinib. Based on the calculation and analysis, we suggest that with other metabolites, reactive electrophiles of Erlotinib: epoxide and quinine-imine, can be generated by several steps along the oxidative reaction pathway. The generation of epoxide needs two steps: (1) the addition of Erlotinib to Compound I (Cpd I) and (2) the rearrangement of protons. Whereas, quinine-imine needs a further oxidation step (3) via which quinone is generated and ultimately turns into quinine-imine. Although both reactive electrophiles can be produced for either face-on or side-on pose of Erlotinib, the analysis of energy barriers indicates that the side-on path is preferred in solvent environment. In the rate-determining step, e.g. the addition of Erlotinib to the porphyrin, the reaction barrier for side-on conformation is decreased in aqueous and protein environment compared with gas phase, whereas, the barrier for face-on pose is increased in solvent environment. The simulated mechanism is in good agreement with the speculation in previous experiment. The understanding of the subtle mechanism of bioactivation of Erlotinib will provide theoretical support for toxicological mechanism of EGFR inhibitors.

## Introduction

Recently, the cancer therapy has shifted from the predominant use of cytotoxic agents and anti-hormonal approaches to molecularly targeted-based design of anticancer agents [1]. The inhibitors of epidermal growth factor receptor (EGFR) tyrosine kinase, have been approved for the treatment of non–small-cell lung cancer (NSCLC) and other solid tumours to inhibit proliferation of cancer cells [2, 3].To date, 14 small molecule kinase inhibitors have been approved for targeted cancer therapy by the Food and Drug Administration. These target-therapy drugs have lower degrees of toxicity, more—selectivity and better tolerance than traditional cytotoxic therapies [4, 5]. For example, Gefitinib, Erlotinib, and Lapatinib have been designed to target the ATP binding pocket of the kinase domain to treat EGFR/HER2-dependent tumours. Although having therapeutics superiorities, EGFR/HER2 inhibitors have been found of life threatening adverse effects in clinic treatment, such as drug-induced hepatitis [6, 7], interstitial lung disease [8–10], and severe skin disorders [11]. The work of Li X, et al suggested that the reactive electrophile of the EGFR inhibitor bioactivated by Cytochrome P450 (P450) might have association with the generation of pulmonary and hepatic toxicities [12, 13].

P450- mediated metabolism is regarded as the most important detoxification pathway. It has become increasingly clear that some xenobiotics can be biotransformed into reactive intermediates and/or metabolites [14–16]. The biotransformation of electrophilic and/or free radical metabolites in bioactivation events is proposed to cause hepatotoxity or other toxicity by covalently binding with essential cellular macromolecules [17]. Experimentally, the fairly successful approach to examine bioactivation is the trapping electrophilic reactive intermediates *in situ*, that is, to identify the compound of interest with liver microsomes or recombinant human P450s in the presence of exogenously added nucleophiles such as N-acetylcysteine glutathione (GSH) and its derivatives [18, 19].The examination of bioactivation potential of promising drug candidates using animal or human reagents may offer some value in toxicity predictions [17]. Generally, from the identification of GSH-conjugate, possible mechanism can be speculated to demonstrate the biotransformation of the reactive electrophilic intermediate. Until now, neither has electrophilic intermediate been caught with experimental techniques, nor has mechanism been proposed on the basis of elaborate theoretical calculation.

As members of monooxygenase class, P450s accomplish the incorporation of an oxygen atom into organic substrate through the complicated catalytic cycle [20]. Series of electron transfer process are triggered after the substrate binds to the heme, leading to two reduction and two protonation steps as well as the formation of a high valent π-cation radical oxo-ferryl species named Compound I (Cpd I) [21–24]. As the active P450 species in the cycle, Cpd I is believed to be the ultimate oxidant that is responsible for the single most important reaction of oxygen incorporation, for example, C-H hydroxylation, C = C epoxidation and sulfoxidation. Cpd I is a tri-radicaloid species with three singly occupied molecular orbitals consisting of two π* anti-bonding Fe-O orbitals and a third that is a combination of a porphyrin π orbital with idealized symmetry and a p orbital on sulfur [25]. The mechanism of aliphatic and benzyl C-H hydroxylation, C = C epoxidation and sulfoxidation catalyzed by Cpd I has been investigated intensively with density functional theory (DFT). For the aromatic oxidation, experiments and theoretical calculations indicated that, different from hydrogen abstraction mechanism, it originates from an initial attack of Cpd I on the π system of the benzene that leads to the production of σ complexes. Proton shuttle mechanism will occurred in the subsequent formation of epoxidation and ketone [26]. In spites of the extensive theoretical investigation on the metabolic mechanism of some general types of oxidation reactions, to date, few investigations have been performed to explore the bioactivation mechanism of EGFR inhibitors.

For one case of the targeted therapeutics, Erlotinib, a second line anti–NSCLC agent, has been found that bioactivation occurs when Erlotinib interacts with cytochrome P450s, and the reactive intermediate can covalently conjugate to the cystein group of the peptide-mimetic GSH [12], which is potentially associated with the pulmonary and hepatic toxicities. The P450 dependent Erlotinib-GSH adducts are proposed to be formed via reactive epoxide and electrophonic quinine-imine intermediates [12]. The reactive intermediate is too active to be caught by experiment approach, and it is of great significance to employ the theoretical method to mimic the bioactivation process catalyzed by P450s. In this context, we focused on the catalytic mechanism of Erlotinib mediated by oxo-ferryl species, Cpd I, ignoring the influence of amino acids. DFT computations were performed to simulate the catalytic process that ultimately produced the bioactive intermediates. The computational simulation of the subtle mechanism of bioactivation will shed light to the exploration of toxicology and provide a helpful tool to avoid -drug adverse effects.

## Experimental

A six-coordinate complex (Fe^4+^O^2-^(C_20_N_4_H_12_)^-^(SH)^-^) was modelled as a Cpd I of P450 and Erlotinib was used as substrate. The model of Cpd I has also been used by other researchers [26–29]. It has been reported that the current model can represent the real P450 enzymes quite well when comparing results between pure quantum mechanics and a combination of quantum and molecular mechanical (QM/MM) method that simulates reactions in the protein environment [30].

All the quantum chemical calculations in terms of the special reaction mechanism—bioactivation mediated by P450s were performed with density functional theory (DFT) using Gaussian 09 program [31]. With the basis set LACVP for iron and 6-31G for the rest, the spin-unrestricted hybrid UB3LYP [32, 33] was employed to optimize the transition states and the stable species (reactants, intermediates and products) without symmetry constraints. With the same method, a higher basis set LACV3P+*(Fe)/6-311+G** (rest) was used to perform the single-point energy calculations. These functions and basis sets have been tested and successfully applied in other researchers’ studies [34, 35]. The analysis of vibration frequencies can denote the stable interspecies and the transition state, since the former possesses positive frequencies and the latter exhibits only one imaginary frequency.

To further verify the transition state, the intrinsic reaction coordinate (IRC) was employed to gain the reaction pathway across the transition state that links the reactive and productive species. To assess the polarity effects on the process of reaction, enzymatic and nonenzymatic environments were mimicked using PCM model in nonpolar solvent (chlorobenzene, ε = 5.62) and polar solvent (aqueous, ε = 78.39), respectively.

Considering that the P450 dependent Erlotinib-GSH adducts were appended upon m-ethyl aniline group of Erlotinib, the original conformations of Erlotinib were designed and optimized with the subgroup of m-Ethylaniline upon Cpd I.

## Results

**Fig 1:**
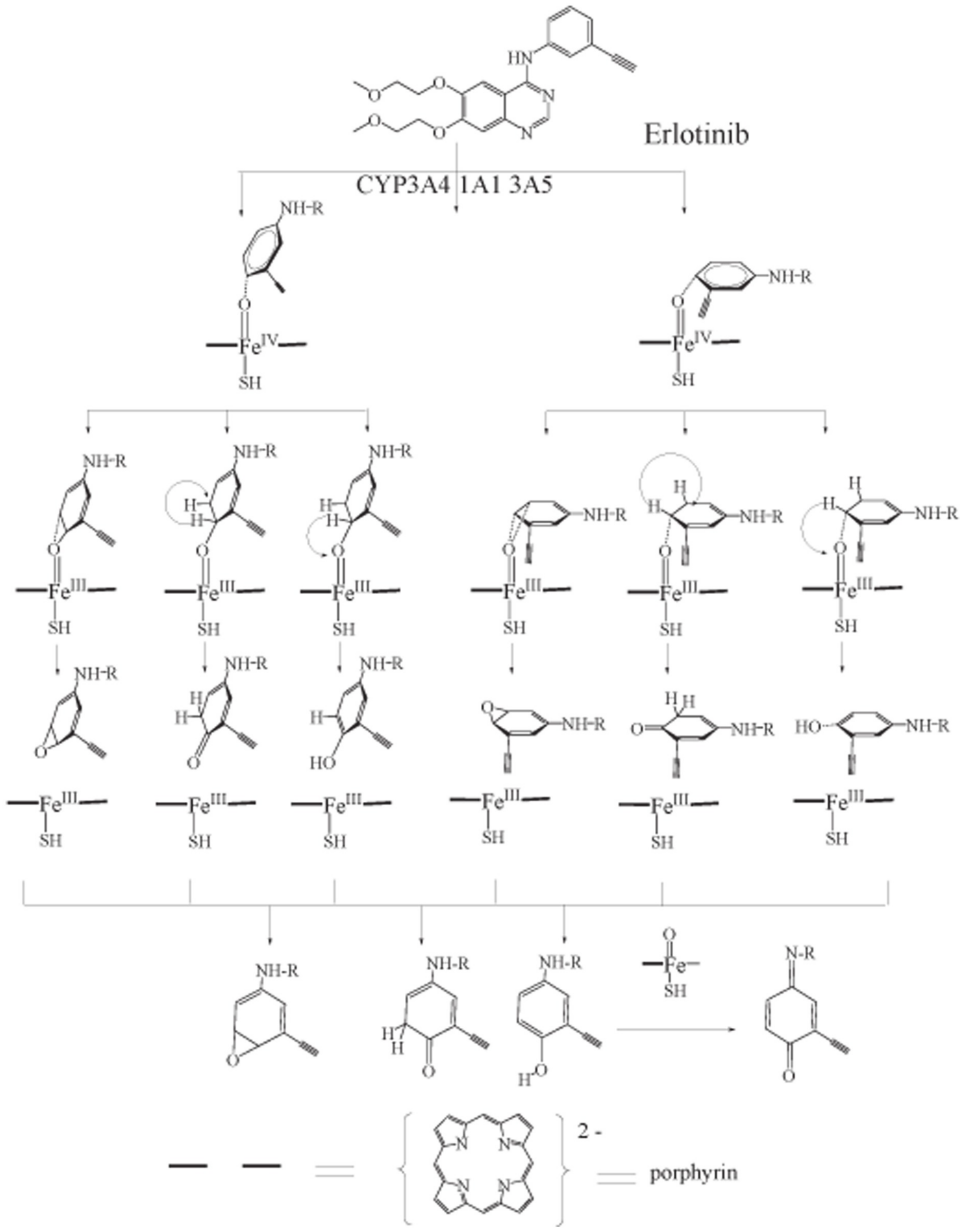
The proposed mechanism for the bioactivation of Erlotinib by P450s.

The bioactiviation mechanism of Erlotinib was proposed as Fig 1. We established two main models: Erlotinib situating its m-Ethylaniline group in face-on and side-on poses upon Cpd I, respectively. The two models were optimized and subsequently submitted to search the reaction pathways of bioactivation. The computation result suggested that both of the face-on and side-on conformations could follow the catalytic pathways to generate the reactive intermediates, e.g. the epoxide and quinone-imine. The attacking of the enzyme or GSH by the intermediate might cause the idiosyncratic toxicities.

### Addition of Erlotinib to Cpd I

**Fig 2:**
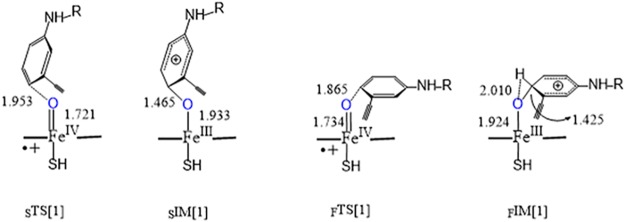
Geometries of the transition state and intermediate for the addition of Erlotinib to Cpd I. _S_ and _F_ denote side-on and face-on poses, respectively.

As shown in Fig 2, Erlotinib was found to situate itself upon Cpd I with face-on and side-on conformation. Based on the initial conformations, the investigation of the subsequent reaction pathways was performed with DFT calculations.

#### Side on

Starting from the initial poses, we optimized the side-on and face-on transition states, which led to the generation of adducts to Cpd I. The pathways along the two kinds of transition states were further verified by IRC method. In side-on path, Erlotinib was added to Cpd I with the para aromatic carbon of aniline group upstandingly towards the active iron-oxo species while with the quinazoline group parallel to the plane, as the geometry of transition state (_S_TS[1]) shown in Fig 2. All of geometries in the context can be referred to the Cartesian coordinates in S1 File. The reactive species Fe = O was appended to the carbon atom of aniline ring to form tetrahedral intermediate (sIM[1]). In this way the length of C…O in sIM[1] turned to be 1.465 Å from 1.953 Å in _S_TS[1], and the distance of Fe = O turned to 1.933 Å from 1.721 Å. It was a slightly endothermic path in gas phase that 16.1 kcal.mol^-1^ energy was needed to overcome the saddle point and 15.4 kcal.mol^-1^ was released in the formation of σ adduct (_S_IM[1]). Solvent medium was favourable to the addition process because the reaction became exothermic both in aqueous and protein environment, with the energy barrier lowered and release energy increased, as shown in Table 1. When it turned to the path of quartet multiplicity, solvation medium shows pimping effect on the energy barrier. Whether in gas or solvation phase, the quartet TS lied a higher energy point than the doublet TS. Anyway, the whole path for quartet state is an exothermic process. The addition process is involved with electrons transferring, from the substrate to the singly occupied orbital of Cpd I, the pophyrin a_2u_ and the π_xz_ orbital. Analysis of the spin densities (ρ) and atomic charge densities (Q) reveals that the transition state of Erlotinib demonstrates a hybrid cationic and radical character. The Q of -0.44 and the ρ of -0.31 in _S_TS[1] suggest there is an excess of β spin density in Erlotinib during the process of electrons transferring to Cpd I, as shown in Table 2. It can be explained as a hybrid anion and radical character that the π electron may first be excited to singly occupied π*orbital, which may be easy to accept the electron transfer from porphyrin a_2u_ and the π_xz_ orbital, subsequently, the electrons are transferred back to the porphyrin orbital of Cpd I.

**Table 1:** Reaction barriers in kcal.mol^-1^ for different reaction section in the bioactivation process of Erlotinib catalyzed by Cpd I. _gas_,_aqu_ and _pro_ represent the gas, aqueous and protein environment, respectively.

Reaction	ΔE _gas_	ΔE _aqu_	ΔE _pro_
addition			
Side on	-16.1	-13.8	-14.6
Face on	-15.4	-17.7	-17.3
epoxidation			
Side on	-0.8	-7.6	-5.3
Face on	-5.8	-12.8	-10.4
NIH-ketone			
Side on	-9.3	-12.4	-11.7
Face on	-1.9	-3.8	-2.7
NIH-phenol			
Side on	2.4	1.1	1.4
Face on	-19.0	-26.0	-23.5
Ketone from phenol			
--	-1.0	-0.4	0.0

**Table 2:** The group spin density (ρ) and charge (Q) distributed on the Erlotinib moiety in the addition to Cpd I, where _d_ and _q_ represent the doublet and quartet state, respectively.

	_S_IM[1]	_S_TS[1]	_S_IM[2]	_F_IM[1]	_F_TS[1]	_F_IM[2]
ρ_d_	0.00	-0.31	0.07	0.00	-0.36	0.03
Q_d_	-0.03	-0.44	0.36	-0.03	-0.24	0.55
ρ_q_	0.00	0.51	0.87	0.00	0.53	0.90
Q_q_	-0.02	-0.14	0.42	-0.02	-0.23	0.55

#### Face on

Alternatively for the face-on adduct, as indicated in Fig 2, the aniline group of Erlotinib parallelly added the para carbon to iron-oxo species, with the quinazoline substructure vertical to the edge of porphyrin plane. In _F_TS[1]. We found that the para carbon of aniline group was added to the Fe = O species in a distance of 1.865 Å and the bond of Fe = O was 1.734 Å, which led to the formation of tetrahedral intermediate (_F_IM[1]) with the C-O length of 1.425 Å and F = O length of 1.924 Å. Different from the side-on path, it was an exothermic path for the face-on addition that needed 15.4 kcal.mol^-1^ to overcome the barrier and 17.5 kcal.mol^-1^ was released subsequently. Also distinct from the side-on path, the addition of Cpd I in quartet spin state turned to be endothermic both in gas phase and solvent phases. The spin and charge densities demonstrated similar changes with those of the side-on one. Erlotinib exhibited negative values of ρ (-0.36) and Q (-0.24) in the transition state, while showed positive charge (0.55) in the upon Erlotinib in the tetrahedral intermediate and the spin density turned to be near zero (0.03), see Table 2.

### Rearrangement pathways to form epoxide and phenoxide

The rearrangement of the tetrahedral adducts was further explored for the formation of the complex of porphyrin ring with Erlotinib epoxide, or phenol and ketone. Only the doublet pathway was considered in the rearrangement. Both the epoxidation and proton transferring processes for side-on and face-on adduct were simulated.

**Fig 3:**
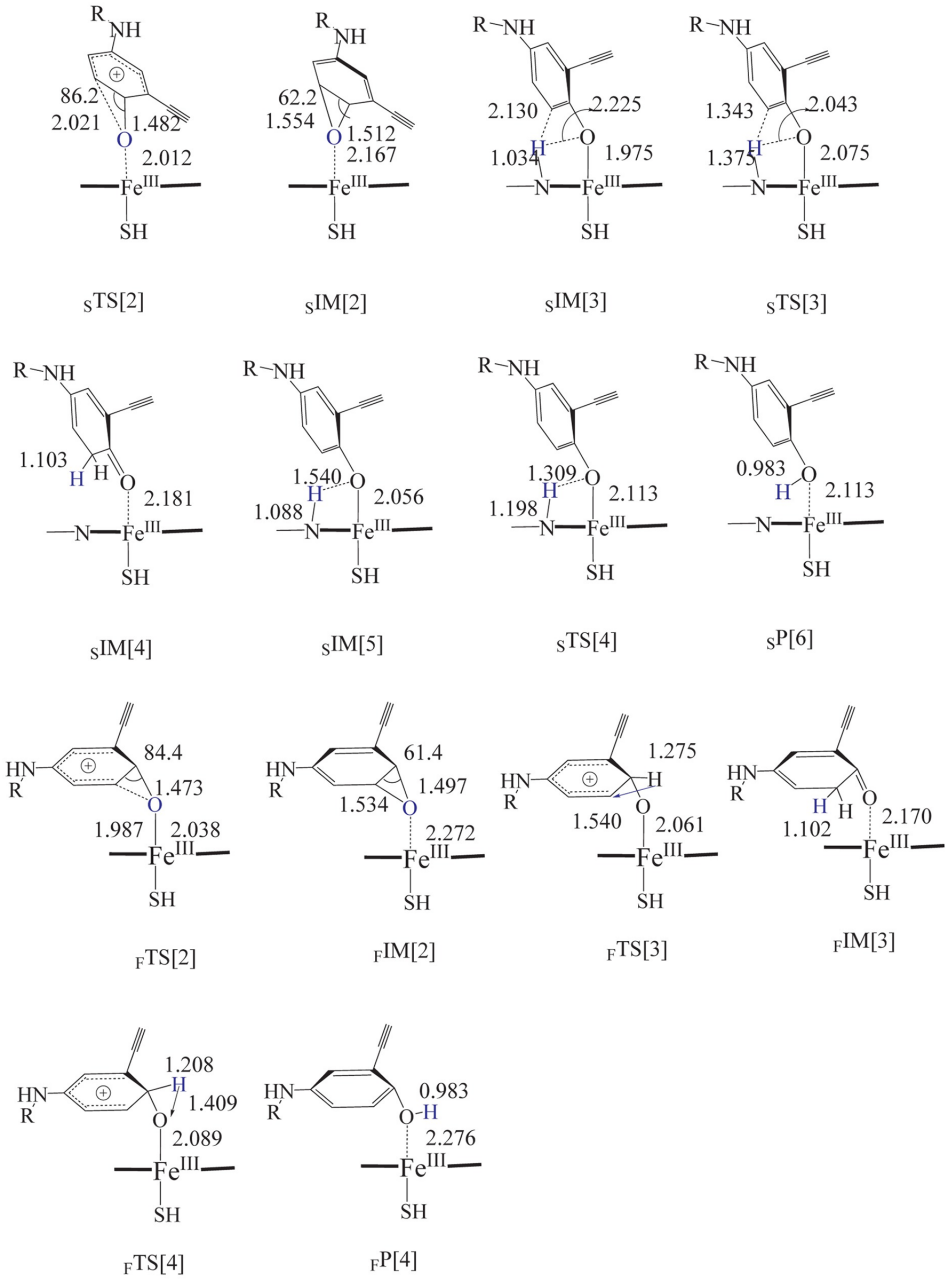
Geometries of the transition state, intermediate and product for the rearrangement of Erlotinib-Cpd I adduct to produce epoxide, ketone and phenol. _S_ and _F_ denote the side-on and face-on poses, respectively.

The epoxidation is associated with the rearrangement of the appended O atom. For the instance of side-on adduct, as suggested in Fig 3, the rearrangement process went from the initial C-C-O angle of 104.8° in the adduct to 86.2° in the transition state (_S_TS[2]), and finally the Erlotinib epoxide (_S_IM[2]) was formed with the angle of 62.2°. Correspondingly, the distance of _α_C-O changed from 2.342 Å across 2.021 Å to 1.554 Å. In such a process, a very small energy of 0.8 kcal.mol^-1^ was used for the arrangement of side-on hybrid cation like adduct to the epoxide and 8.6 kcal.mol^-1^ was let off, which can be found in Table 1. Distinguished from the addition process, the solvation increases the barrier of epoxidation. Barriers of 7.6 and 5.3 kcal.mol^-1^ were computed for the water and protein environments, respectively, much higher than the gas phase. Whereas, the released energies were lowered, which drove the formation of epoxide become an endothermic process. The barrier of the epoxidation from face-on adduct was a bit higher than that from the side-on adduct, which took 5.7 kcal.mol^-1^ in gas phase, while in aqueous and protein the hindrance was increased to 12.8 and 10.4 kcal.mol^-1^ to form the epoxide.

**Fig 4:**
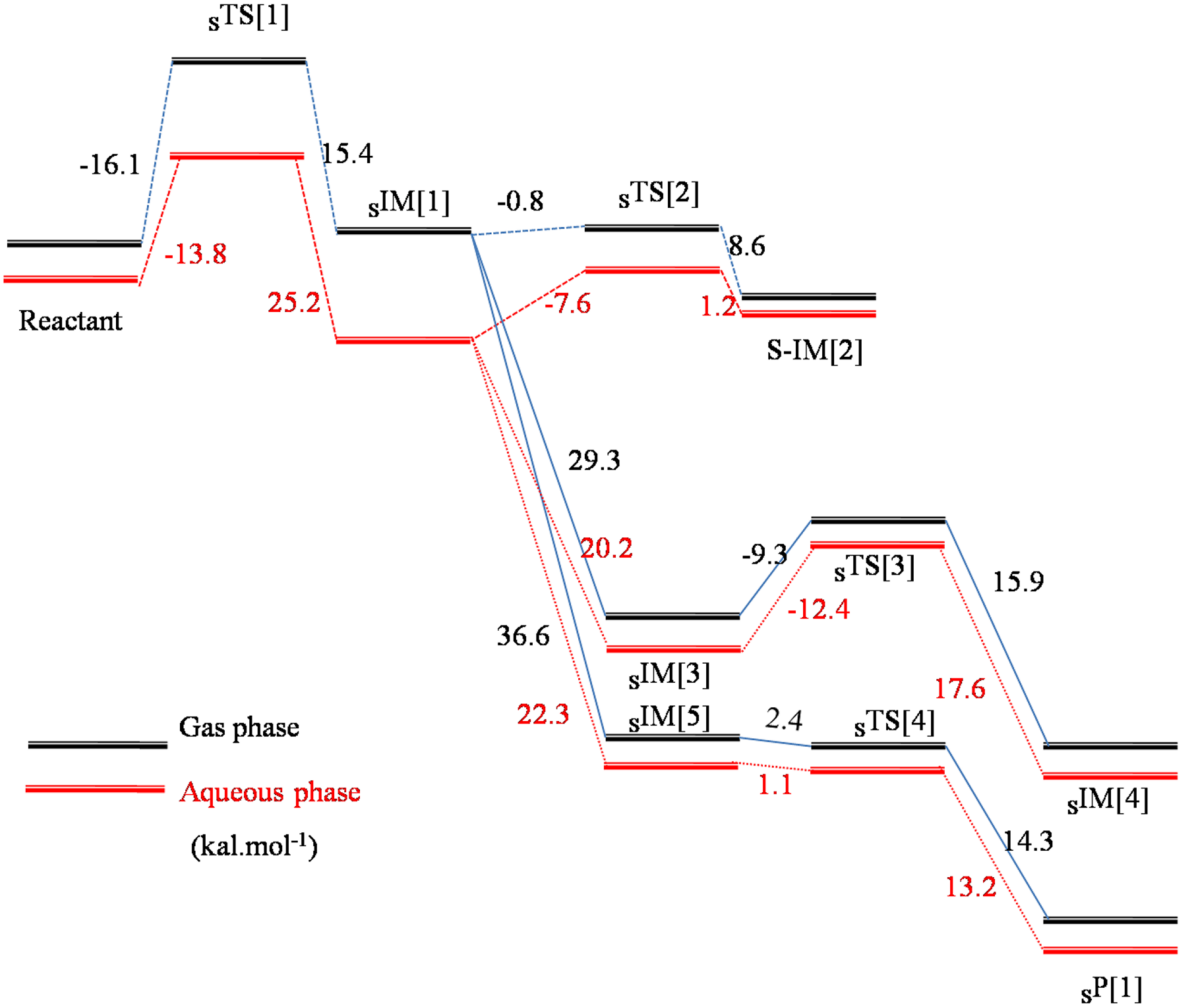
Energy profile (in kcal.mol^-1^) of side-on path for Erlotinib bioactivation by the Cpd I model of CYP3A4 and 1A2 in the gas and solvent phases.

The NIH rearrangement was further considered to visualize the process of migration of proton to the adjacent oxygen or carbon atom. When searching the NIH migrating pathway from the side-on adduct, the proton shuttle mechanism was observed that the ipso-hydrogen was migrated to one of the nitrogen of the porphyrin to form the N-protonated complex first and then transferred to form phenol and ketone, respectively. The energy was lowered significantly when the N-protonated complex (_S_IM[3]) was formed which released 29.3 kcal.mol^-1^ in the ketone pathway and 36.6 kcal.mol^-1^ (_S_IM[5]) in the phenol pathway, respectively. Starting from the N-protonated complex, the way to generate ketone product (_S_IM[4]) needed to overcome a barrier of 9.3 kcal.mol^-1^ via _S_TS[3], whereas a barrierless way occurred to produce the metabolic product of phenol (_S_P[1]) via _S_TS[4]. In the aqueous and protein environment, the barrier turned higher to form the ketone which needed 12.4 and 11.7 kcal.mol^-1^ correspondingly, whereas, the pathway to phenol was still barrierless both in aqueous and in protein environments, see Table 1 and Fig 4. The analysis of barrier energy suggests phenol is much easier to be produced than ketone.

**Fig 5:**
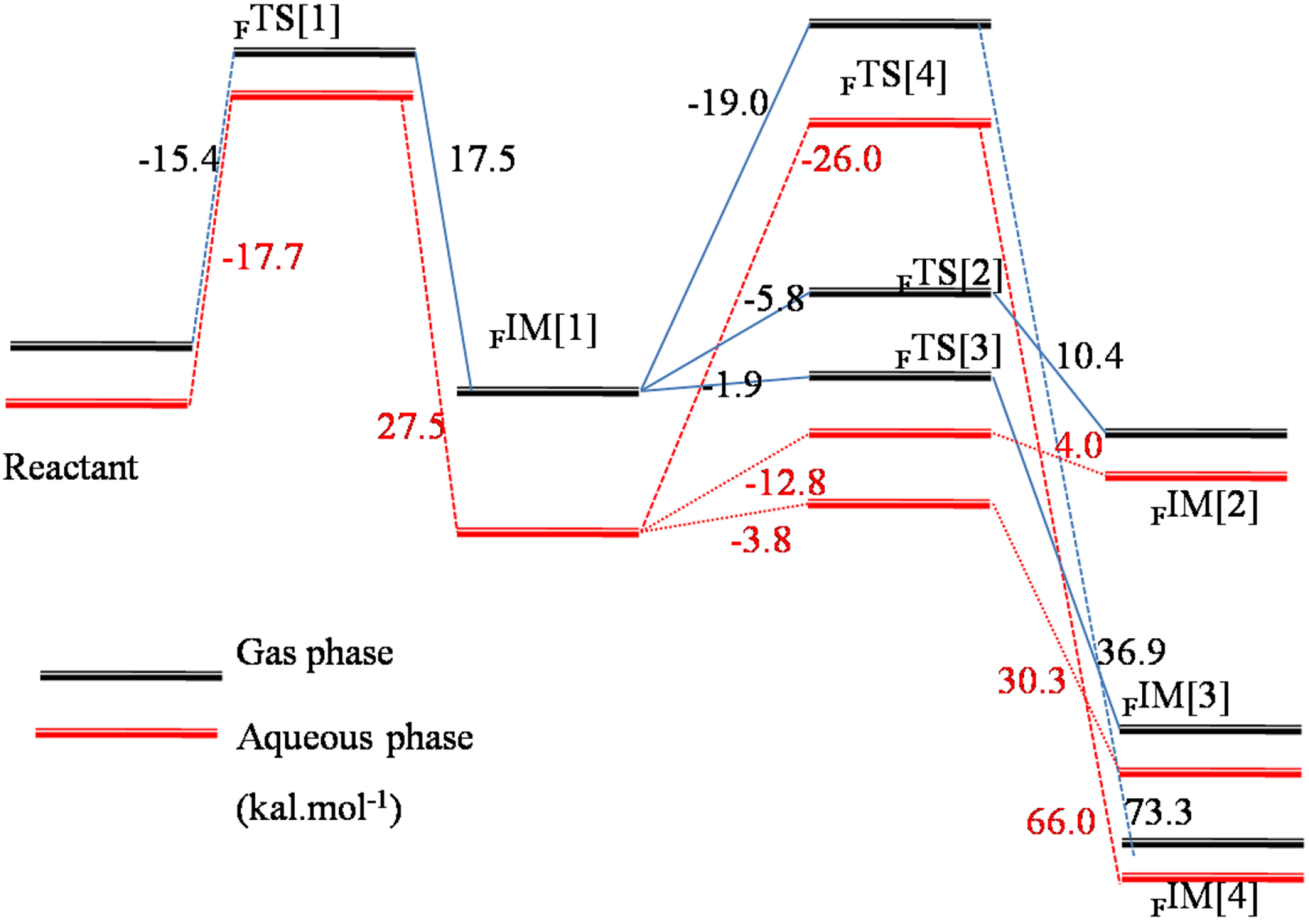
Energy profile (in kcal.mol^-1^) of face-on path for Erlotinib bioactivation by the Cpd I model of CYP3A4 and 1A2 in the gas and solvent phases.

We also considered the possibility to form phenoxide and ketone by rearranging the proton (NIH shift) for the face-on adduct. The transition state was obtained but the shuttle mechanism was not observed. Without the nitrogen in the pophyrin to accept the proton, the ipso-hydrogen migrated directly to the adjacent carbon or oxygen atom, referring the geometry of _F_TS[3] and _F_TS[4]. For the instance of NIH shift to phenoxide, initially, the angle of H-C-O in the face-on adduct was 94.0°, and in the transition state (_F_TS[4]) turned to be 62.6°, simultaneously, the distance of H-O was shortened from 2.010 Å to 1.409 Å. Finally, a phenol metabolic product of Erlotinib (_F_P[1]) was generated. Even without the aid of nitrogen in porphyrin, in gas phase only a very small energy of 1.9 kcal.mol^-1^ was needed to overcome the barrier to form the ketone (_F_IM[3]) and subsequently 36.9 kcal.mol^-1^ was released, whereas, the process of phenyl oxidation illustrated a higher barrier of 19.0 kcal.mol^-1^ to form the transition state in gas phase and a 73.3 kcal.mol^-1^would be let off to form the phenol product, as shown in Table 1 and Fig 5. Similar to the epoxidation, the solvation made the barrier of NIH shift higher that took 3.8 and 2.7 kcal.mol^-1^ for ketone way in aqueous and protein environments, respectively; and correspondingly 26.0 and 23.5 kcal.mol^-1^ for phenol path. As far as the phenol way concerned, distinct from the barrierless shuttle path for the side-on adduct, it needed to overcome a much higher obstacle to reach the product. In addition, comparing with the epoxidation path and ketone path of the face-on itself, the hindrance was also higher, suggesting there was a small possibility for this path to occur. Although we considered the formation of ketone, it should be specified that ketone is not thought to be a reactive intermediate.

### Oxidation of phenoxide to form quinone

**Fig 6:**
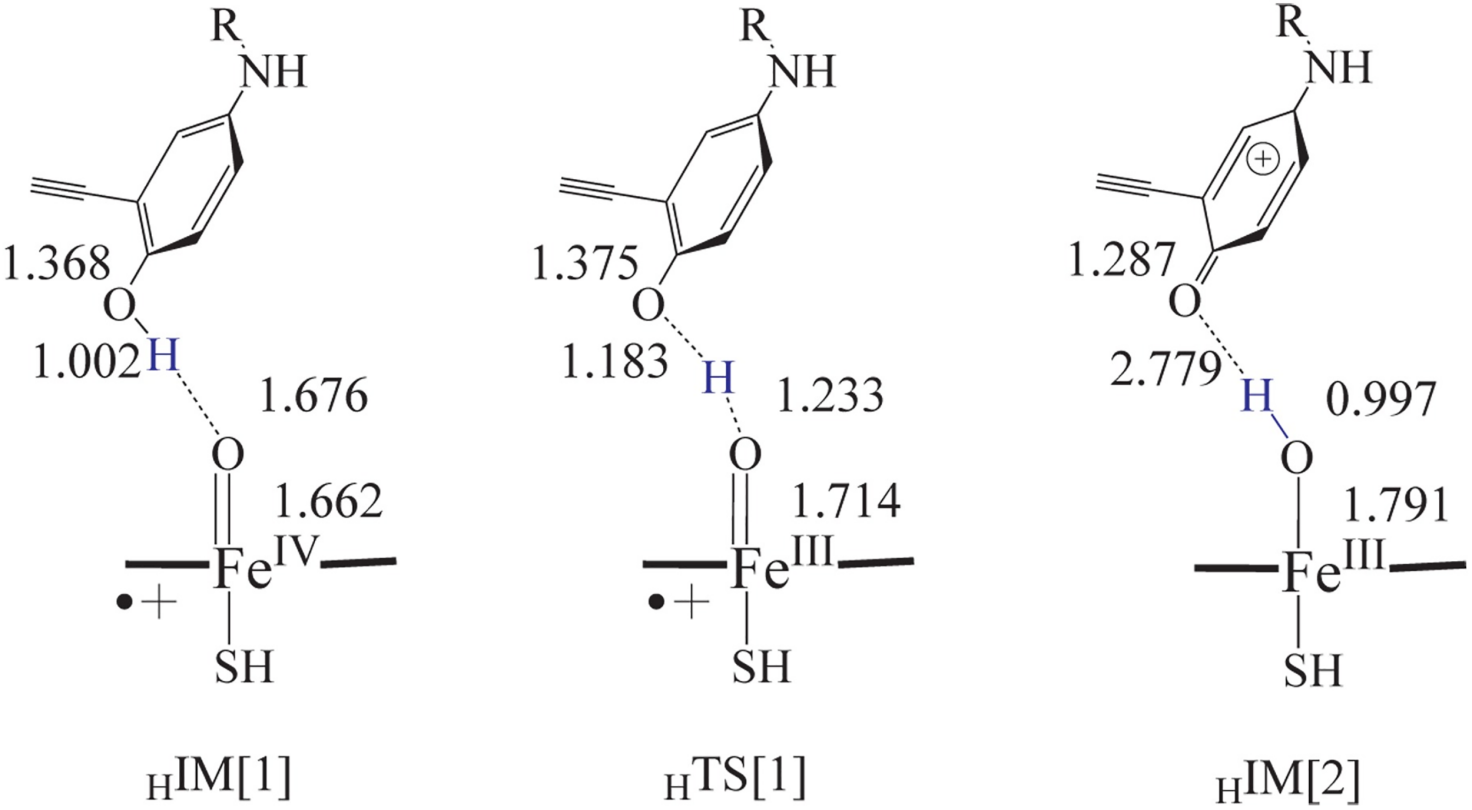
Geometries of the transition state and intermediate for the formation of Erlotinib quinone from phenol catalyzed by Cpd I model.

Whether phenol metabolite of Erlotinib can be further oxidized to quinone was examined to check the route of transferring H atom from hydroxyl towards the active iron-oxo species. With the phenol metabolite adjacent to the porphyrin, we obtained the H-transferring transition state (_H_TS[1]) which resulted in the formation of quinone. In _H_TS[1], the phenyl ring exhibited a side-on conformation bevel to the porphyrin plane with the O-H bond lengthened to 1.183 Å and situated linearly to the = O with a distance of 1.233 Å, as suggested in Fig 6. In gas phase, this path was involved of a marginal hindrance of 1.0 kcal.mol^-1^ and a release of 10.1 kcal.mol^-1^ energies. The influence of solvation is small which makes the barrier even lower, as shown in Table 1.The barrierless way of rearranging H atom to generate phenol metabolite made it of great possibility to form the quinone by a further step of oxidizing the metabolite phenol by the active iron-oxo species, ultimately the intermediate quinine-imine.

## Discussion

Tyrosine kinase inhibitors have emerged as target-based anticancer therapeutics in recent years for the lower toxicity than traditional cytotoxic therapies. Even with the therapeutic advantage, Erlotinib has been found to be associated with idiosyncratic toxicity like interstitial lung disease, liver injury, skin rash, and other life-threatening adverse effects. There has been evidence that the idiosyncratic toxicity may be attributed to the reactive intermediate metabolized by P450s [36] that covalently modifies cellular proteins. In terms of the bioactivation of Gefitinib, a 12-fold increase in GSH adduct formation was detected in human pulmonary microsomes from smokers over nonsmokers, in agreement with P450 1A1 being induced by cigarette smoke. And clinical reports noted an increase in adverse pulmonary events with patients who continued smoking [36]. Although clear mechanism of toxicity has not been established, Erlotinib, another EGFR inhibitor, has been proposed to be in association with the formation of reactive intermediates during the metabolism. However, until now the reactive intermediate has yet been observed in experiment except adducts of nucleophiles like GSH. In this context, a case of EGFR inhibitors, Erlotinib, has been chosen for the investigation of bioactivation mechanism catalyzed by P450s using theoretical method.

The *in vitro* experiment suggests that the bioactivation of Erlotinib was mainly mediated by 3A4 and 1A2 [13]. Although the majority of P450 isoforms show different selectivity on substrate or inhibitor [37–39], 3A4 is well known for its broad substrate specificity. This can be attributed to its vast and flexible active pocket [29, 40]. It provides the basis that we only considered the catalytic process mediated by Cpd I while ignored the influence of amino acids. The poses were in agreement with that of benzene in the mechanism of oxidation mediated by Cpd I, which was proposed by Shaik and Harvey groups, respectively [26, 41]. That’s the reason we addressed the side on and face on as the initial conformations to search the following bioactivation pathway.

And the catalytic process will control the intrinsic reactivity of the substrate with respect to the highly reactive oxygenating species, P450 Compound I (Cpd I) [42]. The electronic and geometric structures of Cpd I firstly came from theoretical calculations and lately were captured and characterized in experiment [24, 25, 42]. Therefore, the model of Cpd I has been used to simulate the mechanism of bioactivation. The catalytic reaction mainly occurred on m-Ethylaniline group for it was situated nearest upon the porphyrin plane. The mechanism of benzene hydroxylation catalyzed by P450s has been elucidated previously which is consisted of initial attack on the π system of the benzene to produce σ complexes and the subsequent rearrangement, through which benzene can be converted to phenol, benzene oxide and ketone [26]. Similarly, Erlotinib is also bioactivated into reactive intermediate of epoxide and ketone through the rearrangement of oxygen and proton upon the addition to Cpd I. Overall, the activation of π-attack is attributed to the rate-determining step. Comparing with the investigation of hydroxylation on benzene put forward by Bathelt et al [41], the face on pose of Erlotinib in doublet state needs a relatively lower energy (Erlotinib: 15.4 kcal.mol^-1^; Benzene: 17.9 kcal.mol^-1^) to form the transition state added to Cpd I, and a slightly higher energy (Erlotinib: 16.1 kcal.mol^-1^; Benzene: 15.6 kcal.mol^-1^) is needed for the side on conformation. Perhaps, this can be attributed to the π—π conjugate of Erlotinib which can lower the energy for the addition in face on style. And similar with benzene, the quartet TS lies a higher energy point than the doublet TS.

Along the reaction path, the reactive electrophiles of Erlotinib can be produced: epoxide and quinine-imine. This is in agreement with the experimental proposition [12]. It has been shown that ERL-G5, e.g. the adduct of GSH to the terminal 3-ethynyl-4-hydroxy-phenyl ring of Erlotinib, was the predominant enzyme-catalyzed GSH adduct [36]. Adducts like ERL-G5 were proposed as the products of GSH adducted to the epoxide or quinine-imine. Either of the two initial adduct complex can go along the pathway to produce the intermediate of epoxide easily, especially for side-on adduct, a barrierless way. In terms of proton rearrangement, there is some difference. The side on can take the mechanism of shuttle bus. So it is very easy to form phenol via proton transfer, which can be further oxidized by Cpd I to generate the quinone intermediate (also barrierless). Additionally, phenol was also a known P450-mediated metabolite of Erlotinib [43]. Without the shuttle mechanism, the face on complex will prefer the way to produce the ketone through NIH shift. However, the ketone is not a reactive intermediate. The solvent environment can, in some sense, influence the pathway. In the aqueous or protein environment, the addition barrier for side on path will be lowered while that for the face on way will be increased, which may explain that the solvation increased the hindrance to generate the π-π stacking complex of Erlotinib and the porphyrin. So do the rearrangement for epoxidation and NIH transfer.

## Conclusions

In summary, computational methods have been employed to specify the catalytic mechanism of bioactivation for a case of EGFR inhibitor, Erlotinib. With the initial optimized conformations, DFT computation was employed to simulate the reaction pathway via which the active intermediate was produced with Cpd I model. The result suggests the bioactivation mainly generates the active intermediate of epoxide and quinine-imine. The epoxide can come through the side on and face on pathways via two steps—the addition of Erlotinib to Cpd I and the rearrangement. While for quinine-imine, it needs several steps by forming the metabolic product phenol first, then being further oxidized into quinone that will easily turn into quinine-imine. The analysis of energy barrier indicates that epoxide and quinine-imine would prefer the side-on path in protein environment. The fact that reactive electrophiles, e.g. epoxide and quinine-imine further covalently conjugate with the biomacromolecules, may act as a potential factor for the idiosyncratic toxicity. This work has illustrated the underline mechanism of bioactivation of EGFR inhibitors, and our data also provide theoretical support for the design of target-based anticancer agents with lower toxicities.

## Supporting information

S1 File Cartesian coordinates of the geometries discussed in the paper. (PDF)
