# Supplementary material for: Computational explanation for bioactivation mechanism of targeted anticancer agents mediated by cytochrome P450s: A case of Erlotinib
Source: PLoS One. 2017 Jun 19;12(6):e0179333. doi: 10.1371/journal.pone.0179333 (PMC5476264; doi:10.1371/journal.pone.0179333)
Supplement: S1 File — (PDF) [file pone.0179333.s001.pdf]

Energy-optimized atomic Cartesian coordinates of the geometries discussed in the paper

$_S$TS[1]
Fe,0,-1.6926600964,-1.1570249908,-0.0218928006
N,0,-3.5297983409,-1.3336069769,-0.8049982961
C,0,-4.6253043089,-1.9906983013,-0.2568940655
C,0,-5.7657528809,-1.860887898,-1.1359666368
C,0,-5.3659960814,-1.1112680996,-2.2054274417
C,0,-3.9753676376,-0.7742580215,-1.9965061672
H,0,-6.7405695213,-2.2831388415,-0.9397410471
H,0,-5.9487217985,-0.7988861923,-3.0597827124
C,0,-3.2205398743,-0.0008711254,-2.8628229827
H,0,-3.7076217489,0.3551906495,-3.7643549628
N,0,-1.1268373377,0.0210677987,-1.5699790235
C,0,-1.8973344366,0.3627379682,-2.6655387644
C,0,-1.1132572666,1.1546152318,-3.5909587785
C,0,0.1346774163,1.2798840489,-3.0523078037
C,0,0.1263273293,0.5694663057,-1.789992644
H,0,-1.4848656758,1.5539813155,-4.5234888632
H,0,0.9906680039,1.8015816235,-3.4551338725
C,0,1.211199827,0.472859318,-0.9324554793
H,0,2.1342550245,0.9550241968,-1.2360144072
N,0,0.1179856066,-0.847005126,0.8356730921
C,0,1.2038962591,-0.186591856,0.2865300467
C,0,2.3348502039,-0.2697331637,1.1887232889
C,0,1.9264274706,-0.9823198213,2.278502879
C,0,0.5412751408,-1.3450710815,2.0548175627
H,0,3.3041450854,0.1671611817,0.9971992134
H,0,2.4940393524,-1.2470931941,3.1586771518
C,0,-0.2313279816,-2.0843664139,2.935630156
H,0,0.2332790649,-2.3975946765,3.864528704
N,0,-2.3121630128,-2.1634039264,1.6069853509
C,0,-1.550660522,-2.4546122898,2.729163126
C,0,-2.340103561,-3.2200349366,3.6700832675
C,0,-3.5779966654,-3.3863237445,3.1170045928
C,0,-3.5603447528,-2.7228577258,1.831121909
H,0,-1.9810362457,-3.5704849845,4.626814435
H,0,-4.4346746743,-3.8983147801,3.5306161729
C,0,-4.6381599099,-2.6515033192,0.9612770571
H,0,-5.5642683069,-3.1265337726,1.2662968647
O,0,-0.9705244359,-2.5172464242,-0.791695525
S,0,-2.4107513483,0.8747630849,1.1265951159

H,0,-1.3687877895,1.697572898,0.7463912245
O,0,-2.4874452241,-10.0876150738,-0.4045510083
O,0,-1.3813469067,-11.9923611587,1.2674435325
O,0,-3.5391374032,-12.296549094,-3.159476154
O,0,-1.5202858972,-14.8082410342,3.551233982
N,0,1.518278693,-6.7882693027,-1.551690284
N,0,2.9619509095,-9.9106016695,0.8062459309
N,0,3.216046314,-7.9423428307,-0.5445092261
C,0,1.0032744551,-8.8890384975,-0.278785687
C,0,1.6086697176,-9.8940900419,0.5439893338
C,0,-1.1272318653,-10.0629314739,-0.1107358482
C,0,-0.5320758466,-11.0200025832,0.7769897483
C,0,-0.3698589989,-9.0265277116,-0.6139711732
C,0,1.8922456044,-7.870275175,-0.7612281784
C,0,0.8127486359,-10.9358813065,1.0793660293
C,0,-3.0615689666,-11.2700841472,-1.0737828474
C,0,0.3731130775,-5.9771608728,-1.5260460957
C,0,-0.8648116135,-12.9698736236,2.2229420223
C,0,3.6876139598,-8.9627011772,0.222682804
C,0,-2.9379213958,-11.1112310908,-2.5824720355
C,0,-2.0444794479,-13.8446277101,2.6080934981
C,0,0.1069970296,-5.1920561566,-2.6664995099
C,0,-0.47345106,-5.8781912571,-0.3939871201
C,0,-0.9738760999,-4.3096509502,-2.6970980264
C,0,-1.5447041402,-5.0055560361,-0.4090421347
C,0,-1.7818565413,-4.138927317,-1.5175409707
C,0,-3.5271360401,-12.3118377527,-4.6075462237
C,0,-2.5095002983,-15.7479083411,4.0385450178
C,0,-1.2691240041,-3.565485123,-3.8730366081
C,0,-1.5334286877,-2.9422099043,-4.8834357287
H,0,2.3175388492,-6.406169132,-2.0457283915
H,0,-0.8614355954,-8.3232978789,-1.2714136266
H,0,1.3125613802,-11.6530979905,1.7159074172
H,0,-2.574524712,-12.1839569833,-0.7331413252
H,0,-4.1090834721,-11.2870783018,-0.7719474111
H,0,-0.0740632035,-13.571417109,1.7618564014
H,0,-0.4601016665,-12.4590352984,3.1030516559
H,0,4.7594025798,-8.9848438083,0.3771912702
H,0,-1.8805394628,-11.0287319822,-2.8793412149
H,0,-3.4592146659,-10.2022414988,-2.9166671545
H,0,-2.4575509068,-14.3444786287,1.7198153037
H,0,-2.8395961935,-13.2357944807,3.0617114466
H,0,0.7357338895,-5.2987086369,-3.544213988
H,0,-0.2693363894,-6.4728989734,0.4870532603

H,0,-2.1842721857,-4.9198489662,0.4605249131
H,0,-2.7383736672,-3.6423331829,-1.6148604153
H,0,-4.0037065082,-13.2461838034,-4.9068858483
H,0,-2.5004620731,-12.28240735,-5.0001797347
H,0,-4.0908885769,-11.4638464211,-5.0221588913
H,0,-1.9860640944,-16.4068857457,4.7319778376
H,0,-2.9349701956,-16.3424808602,3.217669034
H,0,-3.3247215362,-15.2330600793,4.5663960847
H,0,-1.759505125,-2.3895684128,-5.7641025995

$_{S}$IM[1]

Fe,0,-0.5258001159,3.7975765311,0.2547304413
N,0,-2.2608549265,3.3718173373,-0.6831454202
C,0,-3.280111185,2.529004441,-0.2539764676
C,0,-4.3839379425,2.5749575179,-1.189634345
C,0,-4.0423643336,3.4639828753,-2.1691146035
C,0,-2.7234885522,3.9695431151,-1.8478752191
H,0,-5.2990339489,2.0093434639,-1.0875482543
H,0,-4.6228216028,3.7683485014,-3.0283652497
C,0,-2.0406102013,4.9176256399,-2.5955960392
H,0,-2.5331357711,5.2910833602,-3.4879476992
N,0,-0.0144839896,5.0981691866,-1.2049426818
C,0,-0.7857431534,5.4338043913,-2.3007388798
C,0,-0.0775756345,6.3944858205,-3.1267111579
C,0,1.1269360352,6.6251954843,-2.5270619585
C,0,1.1636268824,5.8140282899,-1.3255126154
H,0,-0.467480972,6.8275277747,-4.0370474012
H,0,1.9220569615,7.2826850023,-2.8484494671
C,0,2.2156778036,5.7823620622,-0.422035419
H,0,3.0829040791,6.3972405262,-0.6401990306
N,0,1.2181366031,4.210246937,1.1862071192
C,0,2.2370442372,5.0358490265,0.7476368987
C,0,3.3273039468,5.0278557743,1.7048252364
C,0,2.9582977828,4.1946150961,2.7205539207
C,0,1.640642362,3.6819159654,2.3910374434
H,0,4.2419361181,5.5934437987,1.5989026584
H,0,3.5107036338,3.9391211592,3.6134837552
C,0,0.9280265937,2.7856857286,3.1761019981
H,0,1.3842764332,2.4741264081,4.1104101401
N,0,-1.0510599791,2.514474389,1.7246324823
C,0,-0.3163995529,2.2536741426,2.8723116398
C,0,-1.0425128933,1.3284677372,3.7183640545
C,0,-2.2193596369,1.041191475,3.0851124202
C,0,-2.2261702514,1.7897645037,1.845107999

H,0,-0.6904461474,0.9649593035,4.673227731
H,0,-3.0201344046,0.396623829,3.4183570321
C,0,-3.2587879232,1.7820920762,0.9151124695
H,0,-4.127153748,1.1694541819,1.1347486005
O,0,0.4860729584,2.4684635794,-0.7179932782
S,0,-1.5926774458,5.5326329372,1.4322796913
H,0,-0.5689262778,6.4591995395,1.3849063643
O,0,-1.313518701,-4.8357556124,-0.2108239288
O,0,-0.2327058998,-6.7418016824,1.489600121
O,0,-2.715259182,-7.2115085325,-2.6525600812
O,0,-0.4402848399,-9.3687969698,3.9833112602
N,0,2.7975402861,-1.8669282397,-1.7367157584
N,0,4.1991052236,-5.0028812924,0.6379772075
N,0,4.4880326941,-3.1013506414,-0.7942693235
C,0,2.2440514354,-3.8906342444,-0.353069494
C,0,2.8367338312,-4.9080498375,0.4680298664
C,0,0.0557656454,-4.903646216,-0.0009161845
C,0,0.645121037,-5.8647257046,0.8922979257
C,0,0.8458915089,-3.9421293192,-0.5945078971
C,0,3.1656022724,-2.9579519363,-0.9196338791
C,0,2.0108267531,-5.8692072084,1.0984611547
C,0,-2.0468665793,-6.0106451636,-0.7236142839
C,0,1.8212721454,-0.9149892339,-1.5805728025
C,0,0.2683266093,-7.6981280173,2.4779672739
C,0,4.948768625,-4.1264959407,-0.0210811296
C,0,-1.9658286481,-6.0423278103,-2.2434317209
C,0,-0.9520854378,-8.4233817207,3.0165786753
C,0,1.6553419193,0.016873281,-2.6486830536
C,0,1.0307402515,-0.8002462985,-0.3976855739
C,0,0.7207293942,1.0252047734,-2.5801372006
C,0,0.1012438217,0.1968062548,-0.3004516617
C,0,-0.0816900922,1.2611534646,-1.3233684015
C,0,-2.7880774624,-7.3889803504,-4.0887076004
C,0,-1.4684072892,-10.1589984148,4.6314798406
C,0,0.5287682397,1.9101279107,-3.6664965773
C,0,0.3361835245,2.6594508664,-4.6054457108
H,0,3.5217799046,-1.6549494004,-2.4157692962
H,0,0.3600867684,-3.229815306,-1.2469469579
H,0,2.5005169124,-6.5955913315,1.7322922589
H,0,-1.6576175504,-6.9306359487,-0.2889040434
H,0,-3.0751421666,-5.8610313359,-0.3948004746
H,0,0.9592842271,-8.404165431,2.0052890784
H,0,0.7879778831,-7.1680832174,3.2828421625
H,0,6.024040633,-4.2139261883,0.0653020182

H,0,-0.9182491812,-6.1157056014,-2.5764494825
H,0,-2.3975975601,-5.1256975202,-2.671279106
H,0,-1.4846556162,-8.936574186,2.2026239375
H,0,-1.644254626,-7.7090224387,3.4847665997
H,0,2.2712818107,-0.0921799836,-3.5359945124
H,0,1.2033667461,-1.4744754756,0.4301447768
H,0,-0.4754372892,0.3138081787,0.6071281575
H,0,-1.1415481411,1.4298550487,-1.5632848521
H,0,-3.3755313188,-8.2927751078,-4.2541477791
H,0,-1.7880713893,-7.5165993016,-4.5275304534
H,0,-3.2839192102,-6.5356882786,-4.5728246214
H,0,-0.9491593607,-10.8196675705,5.3262252159
H,0,-2.0291846002,-10.7603197354,3.9021631329
H,0,-2.1712616145,-9.5217123842,5.1861941284
H,0,0.1673888111,3.3351684423,-5.4103608589

$_{S}$**TS[2]**

Fe,0,-0.5940540264,3.5940886302,0.2302089877
N,0,-2.2818725753,3.1147370145,-0.7722765725
C,0,-3.2882080711,2.2423828654,-0.3772075217
C,0,-4.3662940661,2.2635961709,-1.3428067169
C,0,-4.0223331055,3.166837913,-2.3085787201
C,0,-2.7263254902,3.703897048,-1.9496261848
H,0,-5.2679856026,1.6726816095,-1.2685786651
H,0,-4.5856770157,3.459079937,-3.1832413663
C,0,-2.0369569468,4.6557072655,-2.6864545184
H,0,-2.5092646632,5.0191895919,-3.5933204951
N,0,-0.0426357557,4.8497843525,-1.2473150112
C,0,-0.7919324215,5.1778624376,-2.3645908596
C,0,-0.0699959616,6.1388510632,-3.1747246003
C,0,1.1168477337,6.3841218234,-2.5447037269
C,0,1.1309379904,5.5821767383,-1.3387369347
H,0,-0.4395677319,6.5632546831,-4.0974055294
H,0,1.9132629057,7.0481970438,-2.8487926035
C,0,2.1525120256,5.5797888483,-0.4010196598
H,0,3.0176317274,6.2040956006,-0.598992687
N,0,1.1177694725,4.0218843008,1.199836377
C,0,2.141411073,4.8540675244,0.7813450599
C,0,3.1972122444,4.8757817651,1.7750348426
C,0,2.8047437456,4.0539819502,2.7915279669
C,0,1.5068560813,3.5171189557,2.4274700346
H,0,4.1082507252,5.4505659948,1.6901378439
H,0,3.3308473571,3.8190751647,3.7056831241

C,0,0.7857500798,2.6166649321,3.1982943312
H,0,1.2148574696,2.3236501762,4.1510535231
N,0,-1.1286145127,2.2872554923,1.6760725425
C,0,-0.4335649453,2.052940975,2.8517697962
C,0,-1.1666521904,1.1135795494,3.6764032519
C,0,-2.3073634543,0.7875822431,2.9991016736
C,0,-2.2863934263,1.5276844818,1.7541449988
H,0,-0.8410782277,0.7653863992,4.6461988685
H,0,-3.101252142,0.1208516935,3.3038842956
C,0,-3.2844977633,1.4901195201,0.7894309444
H,0,-4.1420873394,0.8526660781,0.977994616
O,0,0.5499046547,2.2251492102,-0.7009686068
S,0,-1.7192802868,5.2916168535,1.3390037395
H,0,-0.6976844963,6.2219911308,1.3499425564
O,0,-0.8836915123,-4.8765299228,-0.6180026388
O,0,-0.0018333769,-6.6422925994,1.349014653
O,0,-3.2386294506,-7.0148551239,-2.3797034204
O,0,-0.411828883,-9.2771094648,3.8097967649
N,0,3.1859061414,-1.6055964651,-1.4577007118
N,0,4.3667503344,-4.5682348626,1.2364710475
N,0,4.7635277529,-2.6830957511,-0.1918861569
C,0,2.5440182459,-3.6423960209,-0.1298696338
C,0,3.0542705478,-4.5873009614,0.8218367973
C,0,0.4130554578,-4.8246489253,-0.1370552489
C,0,0.9013887626,-5.7104785625,0.8826713259
C,0,1.2234830511,-3.8178680881,-0.6189767624
C,0,3.4777466912,-2.6479565962,-0.5584049428
C,0,2.2032212393,-5.5978131519,1.3298772714
C,0,-1.4883655956,-6.1536270057,-1.0384775068
C,0,2.1149916935,-0.7348035587,-1.5180290262
C,0,0.4006781961,-7.553166919,2.4203676788
C,0,5.1534829145,-3.6498666025,0.6871000967
C,0,-2.5921121996,-5.7731729904,-2.009354368
C,0,-0.8258766036,-8.3855088169,2.7495197888
C,0,2.1184695979,0.1697048117,-2.6482893642
C,0,1.0947805961,-0.6709276468,-0.5633745902
C,0,1.1483261994,1.1160498964,-2.8166283122
C,0,0.0707709969,0.2619751071,-0.7156979087
C,0,0.0879020736,1.3009627031,-1.7639237989
C,0,-4.3342509408,-6.8459376971,-3.3116051244
C,0,-1.4625551078,-10.1590507035,4.2779625452
C,0,1.1276482312,1.9721825761,-3.9458128367
C,0,1.0779348201,2.7199307404,-4.9040238985
H,0,4.0226087631,-1.309184961,-1.949708055

H,0,0.8144969527,-3.1696062062,-1.3810617205
H,0,2.6240671493,-6.2662747195,2.0681847137
H,0,-0.7389771576,-6.7840651361,-1.5274012725
H,0,-1.8904587387,-6.6785920375,-0.173086684
H,0,1.2226247787,-8.1940392213,2.0838942344
H,0,0.7244983437,-6.9825700606,3.297088202
H,0,6.199572488,-3.6483693955,0.9652851941
H,0,-2.1746265334,-5.2666670202,-2.8918246786
H,0,-3.3012693662,-5.0869556725,-1.5238906335
H,0,-1.1577351429,-8.9479573157,1.8647026437
H,0,-1.6512572612,-7.7360473987,3.0752340824
H,0,2.9024499509,0.0588949207,-3.3910001641
H,0,1.1119808871,-1.3114076448,0.3070660849
H,0,-0.7478091393,0.302184282,-0.0119242373
H,0,-0.8736463023,1.602799808,-2.1771745928
H,0,-4.7273052224,-7.8448738468,-3.5043398245
H,0,-3.9920645866,-6.3985835373,-4.2558627258
H,0,-5.1281060606,-6.215400567,-2.8863711469
H,0,-1.0186480613,-10.7661366684,5.0674021406
H,0,-1.8246623119,-10.8125220449,3.4718088047
H,0,-2.3099485107,-9.5896554853,4.6850779756
H,0,1.0397691404,3.3894285031,-5.7307374536

$_{S}$IM[2]

Fe,0,-0.7987241163,3.505290428,0.8360411483
N,0,-2.5406007019,2.691079669,0.2070758993
C,0,-3.2878136149,1.6962207099,0.8278184253
C,0,-4.5380418025,1.5075228049,0.125769757
C,0,-4.5576848446,2.4017990887,-0.9072063945
C,0,-3.3168029777,3.1444164075,-0.853231489
H,0,-5.2963783576,0.7907224068,0.4060224078
H,0,-5.3351319897,2.5601775442,-1.6407244995
C,0,-2.9605742199,4.1482819612,-1.7425266903
H,0,-3.6663043926,4.3882256541,-2.5308721664
N,0,-0.7611551089,4.7157156958,-0.7716817224
C,0,-1.7753716341,4.8693144086,-1.707929739
C,0,-1.4089779906,5.8909879247,-2.6660843523
C,0,-0.1747045168,6.3543003702,-2.3086597196
C,0,0.2261952166,5.626263918,-1.1244356351
H,0,-2.0268731008,6.2013699925,-3.4964154483
H,0,0.4186694636,7.1198218822,-2.7874683125
C,0,1.4077055745,5.8349667017,-0.4291518394
H,0,2.0952603478,6.5787898098,-0.8175886412

N,0,1.000101568,4.2125450523,1.3788262323
C,0,1.7625371832,5.178433577,0.7393031849
C,0,2.9794971611,5.4206559838,1.4859440167
C,0,2.9480216298,4.6017874858,2.5777657979
C,0,1.713847111,3.845369924,2.5081390553
H,0,3.7445036203,6.1276452686,1.1991850047
H,0,3.6837398848,4.5025386374,3.3627507099
C,0,1.3333813934,2.8803057943,3.4282735216
H,0,1.9962731669,2.7055391055,4.2691144326
N,0,-0.7755704954,2.1807742736,2.3550587271
C,0,0.1831380341,2.1097808013,3.3545436045
C,0,-0.1846865941,1.0931840102,4.3186398163
C,0,-1.3691450901,0.554900509,3.9040611956
C,0,-1.7421494205,1.2400402313,2.6844412908
H,0,0.3969883003,0.8394731426,5.1931078345
H,0,-1.9518340985,-0.2253057389,4.3721368379
C,0,-2.9082073702,1.00541377,1.9699719988
H,0,-3.5864083778,0.2480359586,2.3485198331
O,0,0.3613513544,2.0861832849,-0.3200747297
S,0,-1.9144190575,5.0454744668,2.1193287968
H,0,-1.1648686608,6.1549069481,1.7754962008
O,0,-0.745521968,-4.584138283,-1.0381562936
O,0,0.1336952601,-6.6105246015,0.648070369
O,0,-2.9973476911,-6.4616452717,-3.1906570579
O,0,-0.2414553188,-9.6456734278,2.6014064605
N,0,3.2749040187,-1.1296737185,-1.24041621
N,0,4.4283464687,-4.4196976371,1.052523854
N,0,4.8248427101,-2.332988691,-0.0662335648
C,0,2.6353303318,-3.3464641111,-0.2471445287
C,0,3.1344565295,-4.4067135057,0.5769403785
C,0,0.5421681493,-4.5676120895,-0.5155473762
C,0,1.0198670283,-5.5868680711,0.3727359279
C,0,1.3392193371,-3.4818931561,-0.8104427396
C,0,3.5529544899,-2.2705405657,-0.4917244464
C,0,2.2978060612,-5.5053781303,0.8901196134
C,0,-1.1961215381,-5.7425629543,-1.8289548286
C,0,2.1260663745,-0.3141118637,-1.2887457357
C,0,0.5382167379,-7.6694461827,1.5705289864
C,0,5.2040054221,-3.4089159311,0.677281404
C,0,-2.510922836,-5.3131759214,-2.4538301379
C,0,-0.6539617717,-8.602080421,1.6884568641
C,0,2.0894988372,0.5936416625,-2.4288483522
C,0,1.0979575236,-0.3513043054,-0.3891533363
C,0,1.0440093774,1.4387079445,-2.6643086395

C,0,-0.0085259309,0.5883019738,-0.5063230413
C,0,-0.0491442096,1.5347231725,-1.6669427789
C,0,-4.2540116109,-6.22737597,-3.8698061353
C,0,-1.2603498778,-10.6463745613,2.844949951
C,0,0.947797788,2.2176076178,-3.850514505
C,0,0.8341662452,2.8871114867,-4.8593233728
H,0,4.136319489,-0.6758654031,-1.5262216628
H,0,0.9395891197,-2.7364771522,-1.4828307762
H,0,2.7135123351,-6.2656822401,1.5369114589
H,0,-0.455403588,-5.975856047,-2.6017966549
H,0,-1.3351342993,-6.6094852653,-1.1836588573
H,0,1.4081051745,-8.2053590595,1.1758781955
H,0,0.7918960497,-7.2425799138,2.5465881539
H,0,6.2375084737,-3.4219336623,1.0010225237
H,0,-2.3597952064,-4.4517215233,-3.1205562308
H,0,-3.2218934085,-5.0181358285,-1.6682510591
H,0,-0.9142380448,-9.0174586081,0.7038793897
H,0,-1.5278225545,-8.0581444353,2.0750154855
H,0,2.8939875544,0.5234330135,-3.1540060315
H,0,1.0859363044,-1.056714862,0.4305754479
H,0,-0.9311787592,0.3683241148,0.0131847366
H,0,-0.996486845,1.9369137057,-2.0019334646
H,0,-4.5050774238,-7.1594713334,-4.3776251451
H,0,-4.1656228691,-5.419547782,-4.6105387612
H,0,-5.0527125141,-5.9711753448,-3.1587475455
H,0,-0.8210942128,-11.3636737429,3.5389341663
H,0,-1.5452339276,-11.1611675675,1.9164482968
H,0,-2.1582582714,-10.2014640065,3.2967108688
H,0,0.7418170102,3.4819845928,-5.7371226128

$_{S}$**IM[3]**

Fe,0,-0.3081674699,2.8866294725,0.9967160295
N,0,-0.5105327648,0.813660478,1.5502461304
C,0,-0.5413269675,0.4002008459,2.9454315341
C,0,-1.3051550989,-0.793568255,3.0314061764
C,0,-1.7965049862,-1.1126387952,1.7785379727
C,0,-1.3864598695,-0.1163495899,0.8562995926
H,0,-1.4931481099,-1.3252512655,3.952851062
H,0,-2.4179389636,-1.9541829067,1.5140386308
C,0,-1.7563960722,0.0419506825,-0.4575900357
H,0,-2.2718312411,-0.7899167791,-0.9209634488
N,0,-1.1524702917,2.4310098002,-0.7625676874
C,0,-1.6304975969,1.2172951617,-1.2090859086

C,0,-2.1554283486,1.3595678659,-2.5552349736
C,0,-2.0110310546,2.6698394906,-2.9046194461
C,0,-1.386584007,3.3419189004,-1.7811509178
H,0,-2.5885169301,0.5550208932,-3.1313737986
H,0,-2.2955477625,3.1525246354,-3.8281035922
C,0,-1.0750340417,4.6901291797,-1.7453211302
H,0,-1.3325268781,5.2799281379,-2.6190875944
N,0,-0.0159241524,4.7716623544,0.4741447491
C,0,-0.4301172705,5.3516172426,-0.7047150024
C,0,-0.0255796897,6.7447342972,-0.7318301938
C,0,0.6548720863,6.9852291369,0.4278538452
C,0,0.6597214222,5.7451578057,1.1796215364
H,0,-0.2307531432,7.4288585543,-1.5422069913
H,0,1.1136555857,7.9060184422,0.7571899941
C,0,1.2467158305,5.5675999317,2.4279315416
H,0,1.7660702065,6.4169826456,2.8593263932
N,0,0.5520153727,3.2364802233,2.793330484
C,0,1.194445492,4.4038470388,3.1775695843
C,0,1.7408067775,4.2483365128,4.5112352074
C,0,1.4080776864,2.9932711702,4.9327776913
C,0,0.667111292,2.3635352532,3.8545753686
H,0,2.2911696557,5.0101375639,5.0438356822
H,0,1.6346139144,2.5221817005,5.8781537415
C,0,0.1091702667,1.0801824458,3.9471894415
H,0,0.1528476178,0.6000239563,4.9195695568
O,0,1.493576207,2.5072984212,0.2808902565
S,0,-2.4108502689,3.3602678501,1.8584600895
H,0,-2.0894067297,4.5056028312,2.5594276211
O,0,-1.2716712407,-3.2651471496,-0.7829054205
O,0,-1.3367691822,-5.8164112453,0.2425172
O,0,-2.8908619703,-3.9627887574,-4.029107843
O,0,-2.9662052461,-8.6854348585,1.7520184276
N,0,3.9194238013,-2.2482886069,-1.4917274482
N,0,3.4580895637,-6.210343583,-0.2851333916
N,0,4.7381568798,-4.3525205416,-1.1135658661
C,0,2.3547063432,-4.0615362957,-0.7725119618
C,0,2.32493512,-5.422790482,-0.3323079981
C,0,-0.0769497676,-3.9860871752,-0.6268068998
C,0,-0.0908000757,-5.3144948995,-0.0955567824
C,0,1.1211787144,-3.373279385,-0.930743378
C,0,3.6588991945,-3.5443572229,-1.1084357291
C,0,1.0917647164,-6.0158150381,0.0329644563
C,0,-2.2568543148,-3.785561267,-1.7515992876
C,0,3.2706078062,-1.0510024949,-1.0400995188

C,0,-1.4232534725,-7.1476495932,0.83635118
C,0,4.5803400628,-5.6415742,-0.7137267616
C,0,-1.8294068411,-3.4660519227,-3.1777607509
C,0,-2.8917878558,-7.3721568324,1.1498486781
C,0,2.9012859189,-0.0668406317,-1.9634510396
C,0,3.0959853448,-0.8017898588,0.3301747867
C,0,2.3198594503,1.1491581004,-1.5558640134
C,0,2.5144163324,0.3904930714,0.7558855892
C,0,2.0783298279,1.3861540153,-0.1614138614
C,0,-2.6410478205,-3.7781835168,-5.4440719412
C,0,-4.3089969962,-9.0858780622,2.1188868869
C,0,2.0092461942,2.1391772178,-2.5344821719
C,0,1.78545285,2.9553068947,-3.4075559973
H,0,4.8692076467,-2.1510981533,-1.8362942192
H,0,1.0938332465,-2.3611515281,-1.3113288088
H,0,1.1291133746,-7.0363180437,0.3889153972
H,0,-2.3955717696,-4.858420311,-1.6137361582
H,0,-3.1876283788,-3.2710247025,-1.5081173207
H,0,-1.0595953918,-7.9045964138,0.1330197431
H,0,-0.8236677396,-7.1935737273,1.7517529757
H,0,5.4734590892,-6.2552872222,-0.7330248396
H,0,-0.8744139377,-3.9566157531,-3.4192827868
H,0,-1.6959062839,-2.3810240252,-3.3083450918
H,0,-3.4925618346,-7.3239060912,0.2298409759
H,0,-3.256935869,-6.5977705546,1.8399597009
H,0,3.0645495531,-0.2439051656,-3.0213733576
H,0,3.437489532,-1.5328263501,1.056156955
H,0,2.451454265,0.6132312974,1.8175862639
H,0,0.4490738294,0.6884208595,1.1837987035
H,0,-3.5051721551,-4.1949150933,-5.9629159047
H,0,-1.7316554376,-4.3074373797,-5.7623930181
H,0,-2.5399024014,-2.7131244016,-5.6980524951
H,0,-4.2199609005,-10.0802714636,2.5575242261
H,0,-4.9678594486,-9.1305053833,1.2400258851
H,0,-4.7446522283,-8.3972685905,2.8568703548
H,0,1.5940761988,3.6812011873,-4.1608055148

$_{S}$**TS[3]**

Fe,0,-0.2680480003,2.8784882207,1.0308798756
N,0,-0.2983562207,0.8286900363,1.4903686309
C,0,-0.2033763828,0.3356881159,2.8302115128
C,0,-0.7826428726,-0.9721095825,2.8880536506
C,0,-1.2985477466,-1.2716831639,1.6479653847
C,0,-1.0716898881,-0.1461835327,0.7930757386

H,0,-0.8132914887,-1.5857768122,3.7770684513
H,0,-1.8060742951,-2.1711948945,1.3334641835
C,0,-1.5222300669,0.0124633825,-0.5011602149
H,0,-1.9617223936,-0.8567856152,-0.9753391763
N,0,-1.1445924989,2.4487443205,-0.7211084183
C,0,-1.5432019295,1.2182288536,-1.2039809561
C,0,-2.1254045368,1.3731478328,-2.5242969513
C,0,-2.0967709809,2.704377562,-2.8209274679
C,0,-1.4865148703,3.3784559914,-1.6911248508
H,0,-2.5155196909,0.5609515927,-3.1203468369
H,0,-2.4523411211,3.2007523574,-3.7121662199
C,0,-1.2816257913,4.7452893558,-1.6085128302
H,0,-1.6172205356,5.3483936123,-2.4458616093
N,0,-0.1372489452,4.8115424831,0.5708358141
C,0,-0.6476264201,5.4082229619,-0.563785561
C,0,-0.3456857817,6.8265812926,-0.5489758063
C,0,0.3742238597,7.0703390765,0.5859570467
C,0,0.5069017245,5.8059944893,1.2816806606
H,0,-0.6392183674,7.5250466865,-1.3191086117
H,0,0.7838722892,8.0082515303,0.9317699852
C,0,1.1781616385,5.6232452697,2.4844029575
H,0,1.6534096306,6.4913117313,2.9291368737
N,0,0.687154003,3.2273264294,2.7681499275
C,0,1.2537517098,4.426293035,3.1790431594
C,0,1.8567843962,4.2618364177,4.4846864197
C,0,1.6272500308,2.9714734792,4.8709229531
C,0,0.895390422,2.3279673774,3.797782438
H,0,2.3680995648,5.0426961757,5.0287260103
H,0,1.9168296378,2.4868623971,5.7920937636
C,0,0.4400062565,1.0069653204,3.8480173446
H,0,0.5755918495,0.4772845465,4.7856564982
O,0,1.5371662335,2.7309804229,0.0191932376
S,0,-2.3352145253,3.1458741741,2.0141819845
H,0,-2.0624268383,4.3151706735,2.6971805994
O,0,-1.3983434688,-3.3204615346,-0.9369896746
O,0,-1.4720118125,-5.8177678641,0.2111683032
O,0,-2.8920802966,-4.2423657409,-4.1852382698
O,0,-3.1387257774,-8.6361402304,1.7739203331
N,0,3.788171793,-2.1937324987,-1.3708304237
N,0,3.3567172902,-6.0939158882,0.0380294517
N,0,4.6382482654,-4.2423190514,-0.7996430061
C,0,2.2312618266,-4.012274525,-0.644952498
C,0,2.2073784294,-5.3482833795,-0.132544472
C,0,-0.2034858492,-4.0045810337,-0.6766887272

C,0,-0.2199245347,-5.3022461389,-0.0721750932
C,0,0.9956636258,-3.3714205771,-0.9287200345
C,0,3.5387704857,-3.4743868199,-0.9162885901
C,0,0.9684276154,-5.9601116761,0.1761889493
C,0,-2.3455115725,-3.9146415944,-1.9032621562
C,0,3.1486977086,-0.9723210339,-0.9782022125
C,0,-1.5705121295,-7.1138384504,0.8784710276
C,0,4.4905066374,-5.5143905154,-0.3404228634
C,0,-1.895022749,-3.6327280057,-3.329967449
C,0,-3.0520928401,-7.356051391,1.1059220393
C,0,2.952919743,0.0279550966,-1.9715828855
C,0,2.8536547476,-0.6853912061,0.3392595278
C,0,2.4513803656,1.291650597,-1.6788726128
C,0,2.2705428894,0.5760261229,0.7018289627
C,0,2.097116508,1.6095864526,-0.3076981727
C,0,-2.6281583197,-4.0800894747,-5.6003848
C,0,-4.4943320829,-9.0463636831,2.0790891486
C,0,2.2953156888,2.2733801069,-2.6999166663
C,0,2.1712626589,3.0985399015,-3.5832966574
H,0,4.7500526167,-2.1058642669,-1.6847839264
H,0,0.9667212593,-2.3804324878,-1.361490764
H,0,1.0069151855,-6.9579339888,0.5914314404
H,0,-2.4448797008,-4.9858720393,-1.727783488
H,0,-3.2979586901,-3.4260548541,-1.6955325675
H,0,-1.1438851452,-7.901189232,0.2477519465
H,0,-1.0334156285,-7.0890980645,1.8325560515
H,0,5.4001228209,-6.0979863105,-0.2620072043
H,0,-0.9014277588,-4.0673510973,-3.5190620333
H,0,-1.8320983551,-2.5487388583,-3.5108438136
H,0,-3.5897626042,-7.3726507715,0.1466304789
H,0,-3.4789906548,-6.5543543229,1.7255823871
H,0,3.2103644867,-0.2120337574,-2.998508155
H,0,3.0855831147,-1.406175699,1.1173917948
H,0,2.5230649847,0.9464574818,1.6998445645
H,0,0.9686945703,0.5565935498,1.0314773294
H,0,-3.4436214862,-4.5827564929,-6.1217447683
H,0,-1.6715999208,-4.5413828734,-5.8846387227
H,0,-2.6103355913,-3.018419453,-5.8856637357
H,0,-4.4139236883,-10.0141307845,2.5751041229
H,0,-5.0959794581,-9.1515174316,1.1651280132
H,0,-4.9876593197,-8.3296236176,2.750828496
H,0,2.0621720594,3.8309098043,-4.3473105159

$_{S}$IM[4]

Fe,0,-0.5851528199,3.4394358955,1.0315226532
N,0,-1.3937078072,1.7083365465,1.6836823219
C,0,-1.1235012482,1.0861546506,2.8954704216
C,0,-1.9740945139,-0.0748440617,3.0513041112
C,0,-2.7608338597,-0.1513485209,1.9362232769
C,0,-2.4108597632,0.9699521717,1.0911502618
H,0,-1.9603273189,-0.7351396451,3.9065409747
H,0,-3.5174549413,-0.8851768809,1.6994677634
C,0,-3.0310456464,1.2812227014,-0.1098464824
H,0,-3.8113439384,0.6138944384,-0.4601762445
N,0,-1.81212246,3.3730730683,-0.5628695417
C,0,-2.7612277095,2.4109382314,-0.868141336
C,0,-3.4651540861,2.7794090595,-2.079655092
C,0,-2.9473373391,3.972249577,-2.4949468199
C,0,-1.9135081532,4.3410378729,-1.5482303804
H,0,-4.2550640823,2.1968213154,-2.5313683776
H,0,-3.2258267637,4.5598775339,-3.3578809803
C,0,-1.1320080112,5.4822424217,-1.6451601344
H,0,-1.3273466826,6.1499706618,-2.4777660522
N,0,0.2958002184,5.1045757711,0.3307125758
C,0,-0.1011895063,5.8256815563,-0.7827402182
C,0,0.7558189422,6.9826027262,-0.9495519616
C,0,1.6774737636,6.9486293601,0.0573988487
C,0,1.3868071315,5.7770720792,0.8578684157
H,0,0.6550026843,7.7108917972,-1.7414425759
H,0,2.4801001362,7.6439953799,0.2566466215
C,0,2.0816599356,5.4058379076,1.999713618
H,0,2.9150211454,6.0288211983,2.3070282022
N,0,0.7290189975,3.4218245628,2.5585193406
C,0,1.7610231412,4.319681386,2.8006274021
C,0,2.4204440351,3.9873138921,4.0447578683
C,0,1.7751981081,2.9002977,4.5639195524
C,0,0.7200001319,2.5471988618,3.6373749196
H,0,3.2572160788,4.5307963386,4.4594251468
H,0,1.9814685995,2.3768967606,5.4865298685
C,0,-0.1458683064,1.4736716707,3.8008456754
H,0,-0.040610126,0.8824491628,4.7046072022
O,0,0.8486522689,2.3995670262,-0.2410324942
S,0,-2.1286475873,4.6546591574,2.2195993726
H,0,-1.2632063124,5.5989331154,2.7396370055
O,0,-1.2186166184,-3.9675262898,-0.9005793993
O,0,-1.0727157334,-6.5386935679,0.1155891164
O,0,-2.7554801401,-4.7284482997,-4.1600431158

O,0,-2.4784516733,-9.6021238527,1.4576919266
N,0,3.8068196962,-2.2549799117,-1.005744929
N,0,3.7535541888,-6.219191752,0.2910065888
N,0,4.8510974863,-4.1928151679,-0.3838994356
C,0,2.432084796,-4.2755660304,-0.4401214267
C,0,2.5363419005,-5.6193416746,0.0424963661
C,0,0.0151385959,-4.5487978452,-0.62229794
C,0,0.1203147364,-5.8664603415,-0.0658299681
C,0,1.1487467518,-3.7829118188,-0.795546105
C,0,3.6768386513,-3.5722503495,-0.5852730108
C,0,1.3623916528,-6.3865830125,0.2410915268
C,0,-2.1192052448,-4.6080735038,-1.877684779
C,0,2.9902561739,-1.1296243171,-0.7164022937
C,0,-1.0556592672,-7.8637431617,0.7315737837
C,0,4.8322412689,-5.4896946705,0.0313449507
C,0,-1.8148985234,-4.0773038768,-3.2711574927
C,0,-2.5079637807,-8.2911874008,0.8470042574
C,0,3.0386039103,-0.0554431585,-1.6915011405
C,0,2.2289088181,-0.9987502265,0.3990122677
C,0,2.3451476785,1.1208609292,-1.5631084079
C,0,1.370209974,0.202480668,0.6231170196
C,0,1.480049237,1.3227957491,-0.3838244623
C,0,-2.6286654456,-4.3227467046,-5.5445939281
C,0,-3.7899272513,-10.1843337127,1.6581926091
C,0,2.4358040794,2.1553630411,-2.5370741537
C,0,2.5155820113,3.0363899751,-3.3691860556
H,0,4.7691944485,-2.0525341463,-1.25749759
H,0,1.0222601639,-2.7951383154,-1.2145526244
H,0,1.4940763243,-7.386821313,0.6304552364
H,0,-2.0248354576,-5.6932959936,-1.8397665746
H,0,-3.1255794247,-4.3244769321,-1.5683734185
H,0,-0.4957780149,-8.5671855577,0.1058659715
H,0,-0.5890516667,-7.8138023724,1.7210902875
H,0,5.7995401224,-5.9549075366,0.1772822445
H,0,-0.778906401,-4.3148589177,-3.5599611632
H,0,-1.9365702827,-2.9844964873,-3.3004894252
H,0,-2.9774469979,-8.3274022694,-0.1468827444
H,0,-3.0681501426,-7.5762081541,1.4665136557
H,0,3.6591613794,-0.2058533321,-2.5700318589
H,0,2.2043536495,-1.77723952,1.1532189433
H,0,1.5567744397,0.6253926253,1.6216261701
H,0,0.3074723091,-0.0889194001,0.6667138803
H,0,-3.3872059998,-4.87942873,-6.0962876459
H,0,-1.6334440224,-4.565883639,-5.9438698511

H,0,-2.8063105416,-3.2443615448,-5.6640270048
H,0,-3.6222742832,-11.1562283845,2.1233483605
H,0,-4.317486562,-10.3207227264,0.7034977811
H,0,-4.4068781345,-9.5616601887,2.3214574924
H,0,2.5772568305,3.8181446728,-4.08898422

$_S$IM[5]

Fe,0,-0.0607435666,0.1041636038,0.0764341802
N,0,-0.229473964,0.1147220302,2.2622502414
C,0,0.8982059406,0.2478863404,3.1225391265
C,0,0.4701565483,0.8835090257,4.3225588585
C,0,-0.871737582,1.1926666551,4.199359155
C,0,-1.3234442956,0.7476666419,2.9242287906
H,0,1.1227540586,1.1274376542,5.1478056058
H,0,-1.4843999385,1.7259838543,4.9115117695
C,0,-2.5787377817,0.8802898858,2.3673756657
H,0,-3.3711486146,1.2394864849,3.0158725888
N,0,-2.0624918565,0.3628221412,-0.0134762964
C,0,-2.922867889,0.6378461761,1.0338527813
C,0,-4.2878269667,0.704572336,0.5409022229
C,0,-4.2449282817,0.4575455576,-0.8000247921
C,0,-2.8522512277,0.2500285871,-1.1453766653
H,0,-5.1514359084,0.9077228375,1.1571941371
H,0,-5.0652819829,0.4201938557,-1.5017000016
C,0,-2.376681316,0.0211709293,-2.4281840653
H,0,-3.1024023207,-0.0093748228,-3.2339009117
N,0,0.0144807686,-0.1705053636,-1.8661470105
C,0,-1.0394207076,-0.1405448035,-2.7659785285
C,0,-0.5287008032,-0.2770193623,-4.1125123601
C,0,0.8324723874,-0.3728973392,-4.0196644523
C,0,1.1707995818,-0.3100822388,-2.6138304079
H,0,-1.1405112551,-0.2899710206,-5.0025838253
H,0,1.5505187698,-0.4837018601,-4.8189804604
C,0,2.4527146049,-0.4483915752,-2.0924954127
H,0,3.2631216895,-0.5890114039,-2.7997351156
N,0,1.8845242339,-0.2691660296,0.298949555
C,0,2.7763284362,-0.4667557883,-0.7453746832
C,0,4.1010343183,-0.7292770837,-0.2178016099
C,0,4.0053549927,-0.6912667935,1.140911752
C,0,2.6224715733,-0.3871533664,1.4673232278
H,0,4.9747218349,-0.9288468321,-0.8202972361
H,0,4.7853974836,-0.8570164099,1.8673124528
C,0,2.1809943313,-0.1266030469,2.7673095693

H,0,2.9243457409,-0.1530307354,3.5576279977
O,0,-0.514845891,-1.8073179499,0.6811677259
S,0,0.3285148695,2.3540290295,-0.0768907934
H,0,-0.7172478238,2.6949135381,-0.9125675217
O,0,5.1634889875,-3.7387946947,2.7037384164
O,0,6.8233366048,-5.4794394352,4.0730541881
O,0,3.4471647494,-2.0612811897,5.4851328837
O,0,9.7312665085,-6.3235925479,6.0729378696
N,0,1.7414587936,-6.9213708333,0.0960667353
N,0,4.5655026691,-9.2623330266,2.073067501
N,0,2.7014845329,-8.9229335915,0.5964794063
C,0,3.7472218223,-6.9811322825,1.6088598161
C,0,4.639891349,-7.8958440862,2.2556711224
C,0,4.9624683464,-5.1224501853,2.6214217826
C,0,5.8349377979,-6.0425042277,3.2814871155
C,0,3.9536804121,-5.5892987833,1.8043735193
C,0,2.728168675,-7.5855090655,0.7838677095
C,0,5.6657797906,-7.4006127075,3.0973668178
C,0,5.0115435917,-3.0871004953,4.013883288
C,0,1.2024854425,-5.6068050176,0.2730773482
C,0,7.7987027299,-6.3574549068,4.7113007381
C,0,3.6211566721,-9.6859165631,1.2393735421
C,0,3.5606945354,-3.1107473882,4.4815584024
C,0,8.7719853519,-5.4460634137,5.4372609246
C,0,0.6636831573,-5.2004105179,1.497111076
C,0,1.135690722,-4.7317683526,-0.8252033916
C,0,0.1032156932,-3.9178603958,1.6550649807
C,0,0.5584429749,-3.4720787746,-0.6980122016
C,0,0.046837437,-3.0240844211,0.5383844667
C,0,2.2279391383,-2.1415996381,6.2690755046
C,0,10.7639867761,-5.6254233799,6.8095695161
C,0,-0.4384881256,-3.5249087168,2.916385628
C,0,-0.9010200686,-3.2031194272,3.9959289683
H,0,1.2218665934,-7.5588510299,-0.4977728353
H,0,3.3372737239,-4.8543156824,1.3081136734
H,0,6.3094996203,-8.1364758852,3.5595833814
H,0,5.6830441133,-3.5427699113,4.7428253807
H,0,5.318770845,-2.0548006358,3.8396715244
H,0,7.3021072223,-7.0314540204,5.4180003684
H,0,8.32200999,-6.9546913714,3.9569955501
H,0,3.5623123251,-10.7525095309,1.0549197242
H,0,3.2948038467,-4.0846103194,4.916882064
H,0,2.8835842425,-2.9130121573,3.6401034965
H,0,8.2423390909,-4.8346219464,6.1827031261

H,0,9.2647606996,-4.7693199721,4.7245091025
H,0,0.6769144983,-5.8784214888,2.3434752518
H,0,1.5466607022,-5.0499787837,-1.7782951197
H,0,0.5011076983,-2.8054837355,-1.5480413243
H,0,-0.4233374117,-0.9003613772,1.9217764737
H,0,2.2616081745,-1.3066284255,6.9710964417
H,0,2.1805390732,-3.0865529194,6.828417812
H,0,1.3373262379,-2.0605064454,5.6323704863
H,0,11.4119714067,-6.3960033997,7.228656487
H,0,10.337702483,-5.0224574681,7.6242089249
H,0,11.3522075864,-4.9700537308,6.1514316802
H,0,-1.3411228449,-2.9445755822,4.9293840471

$_{S}$**TS[4]**

Fe,0,-0.9049048839,3.2532032434,-0.2063064049
N,0,-2.1512500924,2.114498336,-1.5549474375
C,0,-3.0260068988,1.078344018,-1.1412617803
C,0,-4.0857896946,0.965674574,-2.0915411108
C,0,-3.9131406846,1.9477217791,-3.0446887542
C,0,-2.7373445346,2.6879411446,-2.7140355102
H,0,-4.8969545237,0.2560197067,-2.0200065514
H,0,-4.5605088995,2.1694281652,-3.8807672146
C,0,-2.2225773334,3.7839995384,-3.3782242239
H,0,-2.6815434944,4.0485757205,-4.3252032946
N,0,-0.5615434214,4.5652184298,-1.6981964269
C,0,-1.1886797098,4.6086733313,-2.9314225254
C,0,-0.6360233916,5.6929616324,-3.7230884034
C,0,0.3313191556,6.2908095772,-2.9692727462
C,0,0.3749780511,5.585983042,-1.7039223847
H,0,-0.954534553,5.9426051221,-4.7246638845
H,0,0.9621992586,7.1283051247,-3.2288302008
C,0,1.2100371742,5.9064166949,-0.6440526855
H,0,1.8778676573,6.7520526328,-0.7688526352
N,0,0.4824533568,4.1221923583,0.8934013541
C,0,1.2398661251,5.2407828401,0.5735617217
C,0,2.0470946104,5.6215431982,1.7111235696
C,0,1.7628081532,4.742590735,2.7194044211
C,0,0.7852664581,3.8065145108,2.2080163957
H,0,2.732078152,6.4566264804,1.725344683
H,0,2.1722401799,4.7163100926,3.7186661771
C,0,0.2736807915,2.7244323452,2.9141906509
H,0,0.6244819348,2.5844416311,3.9310467079
N,0,-1.1953113218,1.8196053867,1.1532550483

C,0,-0.6221927972,1.7919959897,2.4172587888
C,0,-1.0794703572,0.6224231385,3.1407737544
C,0,-1.9251447844,-0.0539951609,2.3132138707
C,0,-2.006505185,0.6963556376,1.073542931
H,0,-0.7757237436,0.3633458652,4.1442110024
H,0,-2.4578070685,-0.9726009057,2.5067787692
C,0,-2.8651512775,0.3567544383,0.0280923913
H,0,-3.4921759206,-0.5175746546,0.1693731447
O,0,0.245491458,1.9187161379,-1.3731992079
S,0,-2.6071190544,4.5129630175,0.6400750708
H,0,-2.0787918244,5.7670246111,0.4011593077
O,0,-0.2307470647,-3.5708656456,1.6473093855
O,0,-0.0516337375,-6.3371687202,1.6729662433
O,0,-3.0935710063,-3.1637915505,-0.6377626422
O,0,-1.1138370475,-9.6640358913,2.6577707007
N,0,4.2650687141,-1.9508379017,-0.7172896394
N,0,4.3792009504,-6.0916208769,-0.2607034033
N,0,5.3288811696,-3.9635923879,-0.8476658944
C,0,3.0789651931,-4.0209928171,0.0572899927
C,0,3.2252016474,-5.443367854,0.1326890246
C,0,0.8732149028,-4.2182200981,1.0819157097
C,0,1.0144952289,-5.6402925014,1.1279230794
C,0,1.8867701605,-3.4395326942,0.5635958827
C,0,4.1993879114,-3.3087950865,-0.5071287975
C,0,2.1712119605,-6.2309658786,0.6572557377
C,0,-1.5623541395,-3.8083243037,1.0694439721
C,0,3.2125941234,-0.9930240217,-0.8716569774
C,0,0.0573929742,-7.7840738884,1.8283760182
C,0,5.3635249596,-5.3130875543,-0.6974949421
C,0,-1.6702766981,-3.2216424839,-0.3333978124
C,0,-1.2217073097,-8.2279670326,2.5152192884
C,0,2.2099411096,-1.1686932579,-1.8296905955
C,0,3.2199346547,0.1797459269,-0.0970583626
C,0,1.1902102248,-0.2117681648,-1.9997391922
C,0,2.2354235475,1.1497022133,-0.2666775238
C,0,1.1987666388,0.9713475753,-1.202080508
C,0,-3.3778778301,-3.0011510243,-2.0518465113
C,0,-2.2493984156,-10.2718957263,3.3194648695
C,0,0.1804030677,-0.4079735301,-2.9899774177
C,0,-0.6710657024,-0.5797580922,-3.8425860865
H,0,5.1957244303,-1.6690975468,-1.005858353
H,0,1.7431702996,-2.3693697482,0.5749108537
H,0,2.3345753204,-7.2995839472,0.6889681395
H,0,-1.7961518522,-4.8738894498,1.0794844622

H,0,-2.243213731,-3.2827920607,1.7401623523
H,0,0.1572687809,-8.2675818754,0.8503069983
H,0,0.9297836255,-8.0351809571,2.441061006
H,0,6.2922191992,-5.7958135416,-0.9793145306
H,0,-1.1490461563,-3.8518514907,-1.0680911811
H,0,-1.2286687722,-2.2171694564,-0.3658375319
H,0,-2.0972786162,-7.95635753,1.9072502831
H,0,-1.3176998913,-7.7379363867,3.4947635676
H,0,2.2103209518,-2.0543994817,-2.455157808
H,0,4.0001004513,0.3176701657,0.6447031247
H,0,2.2430712896,2.0550628365,0.3263064994
H,0,-1.0137264063,1.7661003739,-1.6954874278
H,0,-4.4652127264,-2.9709106152,-2.1420001523
H,0,-2.987507349,-3.8488120816,-2.6325159473
H,0,-2.9435160623,-2.072333047,-2.4440731993
H,0,-2.0403208997,-11.3414191274,3.3609350546
H,0,-3.179025036,-10.1016988597,2.757601129
H,0,-2.3736338207,-9.882741336,4.3401422876
H,0,-1.3949244829,-0.717368674,-4.6100497225

$_S$**P[6]**
Fe,0,-0.9722256172,3.3172134773,-0.2915113432
N,0,-2.3421307209,2.3251989041,-1.3996497697
C,0,-3.0071571222,1.1483296226,-1.0675701883
C,0,-4.0302542882,0.8691361509,-2.0489691129
C,0,-4.0021952754,1.8865341608,-2.963050768
C,0,-2.9538830442,2.7953338039,-2.5594350628
H,0,-4.6924625475,0.0163080507,-2.0164176336
H,0,-4.6316017012,2.0242034607,-3.8305366935
C,0,-2.5865461644,3.9397050724,-3.2512589509
H,0,-3.1372282408,4.174907303,-4.155968912
N,0,-0.7500620153,4.6646764629,-1.7677928408
C,0,-1.5510468496,4.7914560143,-2.8951470579
C,0,-1.117422173,5.9267284015,-3.6820240378
C,0,-0.0501250547,6.4811061515,-3.0349301871
C,0,0.1721109766,5.7008795195,-1.8368402004
H,0,-1.5758927056,6.2405331592,-4.6086574788
H,0,0.5359913993,7.3412585969,-3.3243693541
C,0,1.1297465693,5.9858351696,-0.8754363887
H,0,1.7812750167,6.8351981078,-1.0508071579
N,0,0.5358371662,4.1854295043,0.7013491967
C,0,1.2801384593,5.2954477947,0.3175186068
C,0,2.2051833158,5.6553165158,1.3693298171

C,0,2.0073846421,4.7760485589,2.3969836683
C,0,0.9698251646,3.8577278484,1.9799347954
H,0,2.9006463672,6.4805347464,1.3186302291
H,0,2.5124326427,4.7373233741,3.3513547349
C,0,0.5272257995,2.77520342,2.7256235386
H,0,0.9619214816,2.6344734879,3.7095884838
N,0,-1.0597821793,1.853873633,1.0763050972
C,0,-0.3973778594,1.8361060694,2.2964108541
C,0,-0.7814468651,0.6610524229,3.0498263228
C,0,-1.6753360363,-0.031812366,2.2862948546
C,0,-1.8618968406,0.7190554701,1.0623482368
H,0,-0.4019585247,0.4089455647,4.0292374279
H,0,-2.1748631195,-0.9598615857,2.5198431019
C,0,-2.756700463,0.3773546441,0.0587465175
H,0,-3.324136177,-0.5401207191,0.1786885007
O,0,0.4752642994,1.9838325071,-1.5642847668
S,0,-2.5743043371,4.5547320752,0.7759162606
H,0,-1.9478457167,5.7837951773,0.6866503554
O,0,-0.2963904416,-3.6291287702,1.745631684
O,0,-0.136057846,-6.4039258423,1.6346027496
O,0,-3.1384073596,-3.2777545788,-0.5726972922
O,0,-1.2565519654,-9.7748715401,2.3752743389
N,0,4.3065618768,-1.926144584,-0.2992927252
N,0,4.3928196465,-6.085446691,-0.0367207398
N,0,5.3798285181,-3.935632113,-0.4531070409
C,0,3.0766487794,-4.0293608511,0.2955798686
C,0,3.2156312328,-5.4543989332,0.3113478117
C,0,0.8208844097,-4.2606995375,1.1970771957
C,0,0.9549120305,-5.6863131794,1.1766102448
C,0,1.8634087562,-3.4644064233,0.7695589304
C,0,4.2291340801,-3.2997496399,-0.1649275031
C,0,2.1320769836,-6.2603001744,0.737146341
C,0,-1.6257601287,-3.8697213768,1.1612108082
C,0,3.2922478846,-0.978949643,-0.6156168132
C,0,-0.0400354156,-7.8586822695,1.7204042301
C,0,5.4072673381,-5.2925869352,-0.3627959115
C,0,-1.7203111691,-3.3018430229,-0.2498474608
C,0,-1.3592262547,-8.3335614235,2.3028532161
C,0,2.3006180919,-1.2511992797,-1.5650690993
C,0,3.3389985683,0.2923721304,-0.0148568363
C,0,1.3316767636,-0.2822186676,-1.8902094548
C,0,2.405377359,1.2726033964,-0.3452050336
C,0,1.3978552829,0.9850685558,-1.2651148372
C,0,-3.4140777476,-3.0883221809,-1.9840676914

C,0,-2.4319156706,-10.4182631459,2.9255358186
C,0,0.3155102794,-0.5343313063,-2.8613605412
C,0,-0.561879281,-0.6915987962,-3.6896448593
H,0,5.2655552218,-1.6264701228,-0.4369396809
H,0,1.7250099403,-2.3946736811,0.8334016503
H,0,2.2903085088,-7.3300114965,0.728805872
H,0,-1.8632184098,-4.9334310085,1.1823884388
H,0,-2.3072534277,-3.3310299447,1.8204095635
H,0,0.1190181837,-8.2895418752,0.7258231387
H,0,0.7928306333,-8.1448986707,2.3713305015
H,0,6.3541485203,-5.7635409645,-0.598764915
H,0,-1.175427246,-3.9317901519,-0.968456511
H,0,-1.2981733887,-2.2889386341,-0.2860448769
H,0,-2.1957284948,-8.0288937205,1.6568137821
H,0,-1.5164538297,-7.8957022894,3.2990148773
H,0,2.2725260183,-2.2122592799,-2.063830731
H,0,4.1072450199,0.501875504,0.7216066104
H,0,2.4331045136,2.2522494379,0.1122414641
H,0,-0.2107576857,1.6846751246,-2.2015094158
H,0,-4.5003637717,-3.1073487174,-2.0863990842
H,0,-2.9765698181,-3.898414388,-2.5848163968
H,0,-3.0242240772,-2.1266922168,-2.3430631114
H,0,-2.2204171533,-11.4880442618,2.9251553851
H,0,-3.3216420289,-10.2201611751,2.3109214224
H,0,-2.6269694997,-10.083332468,3.9541809671
H,0,-1.3215633815,-0.8301555698,-4.4224110231

$_{F}$TS[1]

Fe,0,-1.6717972947,-0.9727573199,-0.1892139419
N,0,-3.6159213023,-0.3990266698,-0.0383604851
C,0,-4.5419245208,-0.8827600764,0.8672161758
C,0,-5.8421805965,-0.3020234605,0.5972900985
C,0,-5.6940598802,0.5294335173,-0.4750939022
C,0,-4.3021511711,0.4652887124,-0.8739352419
H,0,-6.7370266272,-0.5121421176,1.1650051633
H,0,-6.4433972747,1.1363443262,-0.962361525
C,0,-3.7489163407,1.1706123888,-1.9315868351
H,0,-4.4074928943,1.8182526723,-2.50053922
N,0,-1.4364433673,0.3427851973,-1.7161303661
C,0,-2.4198959991,1.1085588686,-2.3215012675
C,0,-1.8518362228,1.8438304213,-3.4331565231
C,0,-0.5293853091,1.5118320339,-3.4992581332
C,0,-0.2736876147,0.5712883679,-2.4273414309

H,0,-2.4070403117,2.5193158685,-4.0678140607
H,0,0.2168497984,1.8626286409,-4.197356497
C,0,0.9582871324,-0.0081988,-2.1635445074
H,0,1.7891260736,0.2732625498,-2.8016905287
N,0,0.2900124787,-1.3920181534,-0.2295802417
C,0,1.2179766546,-0.90323142,-1.1388111124
C,0,2.5251508387,-1.4511672632,-0.8455003907
C,0,2.3883466253,-2.2554234079,0.2512783025
C,0,0.9934640384,-2.2117858686,0.6362029744
H,0,3.4224857128,-1.2231881288,-1.4025706263
H,0,3.1489316681,-2.8142264279,0.7784978239
C,0,0.4562130889,-2.8934094538,1.7181536575
H,0,1.1273879811,-3.5091038664,2.3073010274
N,0,-1.8653358959,-2.1190275017,1.4466581796
C,0,-0.8762569264,-2.8424148488,2.0941915579
C,0,-1.4414422201,-3.5458875245,3.22513248
C,0,-2.7721860514,-3.2410033809,3.2620473423
C,0,-3.0358770226,-2.342506634,2.1581120746
H,0,-0.8779110385,-4.1767517255,3.8972016724
H,0,-3.5166170701,-3.5728067525,3.9712964203
C,0,-4.2751207051,-1.7854161967,1.8846676157
H,0,-5.0998152041,-2.066733624,2.531107694
O,0,-2.2058075777,-2.1488975784,-1.3455827133
S,0,-1.1708418183,0.8744276901,1.3251882881
H,0,-1.867684768,1.8670444342,0.6640255431
O,0,-1.769939102,-9.9916962136,-0.8137154863
O,0,-1.4098456979,-11.4765016431,1.5074458337
O,0,-2.3689424813,-12.6374809463,-3.2383122631
O,0,-2.1360101674,-14.1970810191,3.7958155177
N,0,1.3204217164,-5.7599145586,-0.2341440931
N,0,1.8931467612,-8.2828663655,3.0368866582
N,0,2.3899632643,-6.3424464349,1.7102720936
C,0,0.6882826608,-7.8800845641,0.931388669
C,0,0.9462313533,-8.6570226548,2.1065338521
C,0,-0.8645838928,-9.5900938902,0.1573723157
C,0,-0.6618005722,-10.3234609414,1.3728408808
C,0,-0.2031342072,-8.3966081552,-0.0440566981
C,0,1.438909768,-6.6634162398,0.8170059802
C,0,0.2406597371,-9.8664614053,2.3140125508
C,0,-1.734975099,-11.3665443107,-1.3420218125
C,0,0.1706356881,-5.2376410975,-0.8484449464
C,0,-1.2846698584,-12.2691392865,2.728788304
C,0,2.578135211,-7.1762389668,2.7724409006
C,0,-2.3308948961,-11.2792342739,-2.7358993119

C,0,-2.2600273083,-13.4232966031,2.5800781293
C,0,0.3479947616,-4.5124427925,-2.0430493615
C,0,-1.1249282728,-5.3469532213,-0.2783726491
C,0,-0.7235990415,-3.8685403763,-2.6608758925
C,0,-2.194375235,-4.7126720874,-0.873500307
C,0,-2.0251597765,-3.8760509754,-2.026071891
C,0,-2.8956503328,-12.7466270781,-4.5823819212
C,0,-3.0015296896,-15.3582497062,3.8399886315
C,0,-0.5487798136,-3.1851522718,-3.8946133652
C,0,-0.4085653084,-2.606797258,-4.9556691283
H,0,2.1475360443,-5.1780211373,-0.3166415818
H,0,-0.3855611754,-7.873199633,-0.9724644458
H,0,0.44893104,-10.4041770347,3.2287577824
H,0,-0.7036139923,-11.7312316472,-1.383163552
H,0,-2.3232256464,-12.0255126589,-0.704227446
H,0,-0.2601917818,-12.6410966734,2.8372071837
H,0,-1.5399324723,-11.6596300917,3.6021314672
H,0,3.3542864622,-6.8878372481,3.4706952094
H,0,-1.7131380607,-10.6376139709,-3.3811280142
H,0,-3.3410193637,-10.8468414339,-2.6890003628
H,0,-2.0060991303,-14.0285886104,1.6976936376
H,0,-3.2839360404,-13.0426759643,2.4540320332
H,0,1.3328248796,-4.4644747196,-2.4945057384
H,0,-1.2667325992,-5.9036973832,0.6384228031
H,0,-3.181162368,-4.7839913794,-0.432112198
H,0,-2.8845723787,-3.6822955995,-2.6543247868
H,0,-2.8661834636,-13.8069028457,-4.8366818185
H,0,-2.2838781363,-12.1810805135,-5.2999566011
H,0,-3.9327227515,-12.3855693405,-4.6377649551
H,0,-2.8101908715,-15.8428514954,4.7978869661
H,0,-2.7743432778,-16.0575816561,3.022926984
H,0,-4.0605813313,-15.070081219,3.7795483024
H,0,-0.2919058196,-2.0922988951,-5.8798665998

$_{F}$IM[1]

Fe,0,-0.4960384907,3.6446872186,-0.314129256
N,0,-2.5110348147,3.6701495049,-0.14752756
C,0,-3.258894086,2.9471324138,0.7589596521
C,0,-4.6734449124,3.1890415295,0.5383570799
C,0,-4.7695602579,4.0589142819,-0.5092623795
C,0,-3.4155681998,4.3549981701,-0.9376141196
H,0,-5.4705602428,2.746549697,1.1184917508
H,0,-5.6610378247,4.4707753243,-0.9601096734

C,0,-3.0815400893,5.2019545483,-1.9851202592
H,0,-3.8955056247,5.6796877725,-2.5210275431
N,0,-0.6380540353,4.9640344739,-1.837231123
C,0,-1.7889036795,5.4808814467,-2.4042885342
C,0,-1.445084486,6.3610711946,-3.5052791626
C,0,-0.0834417529,6.3653130731,-3.5991696921
C,0,0.4154461268,5.4868346494,-2.555915086
H,0,-2.1617746132,6.8958751852,-4.1119116242
H,0,0.5391588688,6.9069616878,-4.2969683736
C,0,1.7623757924,5.2243109063,-2.3263602882
H,0,2.4799921688,5.7417289988,-2.9555159025
N,0,1.5223900318,3.6320422805,-0.4557957166
C,0,2.2721653436,4.3912154794,-1.3431706138
C,0,3.6840771938,4.2047910194,-1.0777807645
C,0,3.7843623772,3.3572505338,-0.0108585051
C,0,2.4342188487,3.0130249694,0.3845538709
H,0,4.4803689985,4.6823969151,-1.6306728948
H,0,4.6784734894,3.0089658767,0.4867055571
C,0,2.1047425634,2.2004688722,1.4606231873
H,0,2.9184416256,1.7937420397,2.0526975862
N,0,-0.3417167451,2.3467249383,1.2305596365
C,0,0.8082132065,1.9100247168,1.8666940736
C,0,0.4627344823,1.0933013481,3.0103465731
C,0,-0.9026293833,1.0544076947,3.0780662021
C,0,-1.4072561883,1.8544455385,1.982218853
H,0,1.179751689,0.6328640788,3.6751364765
H,0,-1.5221291703,0.5577074471,3.8112485664
C,0,-2.7484911685,2.1105236869,1.746243401
H,0,-3.465532994,1.6355036685,2.4087449948
O,0,-0.7762161948,2.3006331007,-1.6620700974
S,0,-0.3420627141,5.4156844056,1.2347975098
H,0,-1.3128886768,6.2353813294,0.6920890735
O,0,-0.6288251543,-5.1312445205,-0.7015917344
O,0,-0.491506355,-6.7085355749,1.5953812993
O,0,-1.7006354999,-7.6462321136,-3.0981638264
O,0,-1.5628494879,-9.3640263181,3.8238318716
N,0,2.9278907037,-1.3084398908,0.031269854
N,0,3.1570073362,-3.983884141,3.2293282037
N,0,3.8968691295,-2.0848904984,1.9638864549
C,0,2.0353843276,-3.3839501741,1.1261005318
C,0,2.1843109615,-4.2183382223,2.2836851167
C,0,0.2995486168,-4.8762021327,0.291171605
C,0,0.3947479298,-5.6574719048,1.4930451113
C,0,1.1035499698,-3.7669856074,0.1273931288

C,0,2.9226483865,-2.2652444613,1.0663404785
C,0,1.335234515,-5.3375892274,2.4529177726
C,0,-0.8541631571,-6.4964078892,-1.2111251346
C,0,1.8963302598,-0.5950125838,-0.536138711
C,0,-0.4822137742,-7.5352554166,2.802283106
C,0,3.9756150207,-2.9622474129,3.0064002831
C,0,-1.3962687978,-6.3146209156,-2.6178848267
C,0,-1.5875855902,-8.5606582348,2.6220297297
C,0,2.2229353152,0.2553229121,-1.6297560561
C,0,0.5502435394,-0.6707518051,-0.0574456585
C,0,1.2674333594,1.02792192,-2.2543840575
C,0,-0.4103851576,0.113210812,-0.6222247989
C,0,-0.1799597003,1.0128258553,-1.7926119237
C,0,-2.2411324443,-7.6718666303,-4.4417614929
C,0,-2.5691861276,-10.4068834241,3.8537866859
C,0,1.5819371905,1.7986616961,-3.3953978451
C,0,1.8275409329,2.4526577902,-4.3919939959
H,0,3.8581921389,-0.9311683822,-0.1175105252
H,0,0.9967983597,-3.2046219762,-0.7898121426
H,0,1.464218197,-5.9183624828,3.3556990549
H,0,0.0855267078,-7.056911468,-1.2232247869
H,0,-1.5734870389,-7.0150465072,-0.5789186017
H,0,0.4878674784,-8.031178885,2.9138441331
H,0,-0.6733432283,-6.9145942095,3.6838652045
H,0,4.773779276,-2.7891303266,3.7167322116
H,0,-0.6514751562,-5.8233389446,-3.2609917615
H,0,-2.2981063937,-5.6856005741,-2.5955870438
H,0,-1.4002142933,-9.1747572725,1.7292481161
H,0,-2.5581592933,-8.0588904853,2.4985034326
H,0,3.2519626703,0.2890680284,-1.9740003971
H,0,0.3238880715,-1.2776241466,0.8079790399
H,0,-1.4221843711,0.1089058611,-0.2351156274
H,0,-0.7372509199,0.5434688376,-2.636833451
H,0,-2.4266748005,-8.720831686,-4.6756463213
H,0,-1.5289309709,-7.2550719568,-5.1682054363
H,0,-3.1837693522,-7.1097359206,-4.504496948
H,0,-2.4394567397,-10.9250770017,4.8042901935
H,0,-2.4332204043,-11.1175433225,3.0264911121
H,0,-3.5828130938,-9.985319046,3.8007544367
H,0,2.0258594471,3.0298119649,-5.2639349387

$_F$TS[2]

Fe,0,-0.3058767499,3.6263901836,-0.1693220313

N,0,-2.2855729736,3.5511193976,0.2307667824
C,0,-2.8737963463,2.9372950263,1.3217344325
C,0,-4.3143310194,3.091628223,1.2566510047
C,0,-4.5906204998,3.7883163263,0.1150473915
C,0,-3.3214204562,4.0722834172,-0.5245602809
H,0,-5.0066370502,2.7079078412,1.9923328152
H,0,-5.5538487397,4.0910222062,-0.2699685122
C,0,-3.1714549309,4.7761411741,-1.71138926
H,0,-4.0718355449,5.1339660882,-2.2000699353
N,0,-0.7095370246,4.7164915998,-1.8147011659
C,0,-1.9563330302,5.0801640997,-2.3047229274
C,0,-1.7991806179,5.8794760083,-3.5022704999
C,0,-0.4578743714,6.0120036189,-3.7220992856
C,0,0.220628557,5.2859153721,-2.6671479068
H,0,-2.6168547161,6.2877482332,-4.0788196238
H,0,0.0413640296,6.548518818,-4.5162397226
C,0,1.5994720806,5.1802285689,-2.5447172066
H,0,2.2058169413,5.7079451949,-3.273572948
N,0,1.6645347212,3.6940055718,-0.5765160236
C,0,2.2652003536,4.4498194976,-1.5728656783
C,0,3.7058550446,4.3391816998,-1.4727690643
C,0,3.9745113283,3.5215584051,-0.4121566086
C,0,2.7001865502,3.1315889326,0.1552278354
H,0,4.4055726605,4.8323210604,-2.1322047085
H,0,4.93773694,3.2219700151,-0.0239264461
C,0,2.5446205999,2.3323574354,1.2778331594
H,0,3.4417338073,1.9491559411,1.7541762995
N,0,0.0935770017,2.4806022763,1.4401974967
C,0,1.3270158843,2.0412902983,1.8817793944
C,0,1.1735520994,1.2659239876,3.0964548741
C,0,-0.159424518,1.2567231956,3.3949609079
C,0,-0.8333192771,2.0196675964,2.3625321737
H,0,1.9879725768,0.8004548031,3.6318808932
H,0,-0.6550100281,0.7888356833,4.2336504025
C,0,-2.2016520824,2.2368732068,2.3143772574
H,0,-2.800120791,1.8109856271,3.113443924
O,0,-0.7669368366,2.0015584926,-1.3105058777
S,0,-0.0821942171,5.5396377764,1.1210697097
H,0,-1.2627997885,6.158815473,0.7568234305
O,0,-0.6458837244,-5.1443016827,-0.8209386198
O,0,-0.7465458317,-6.6444440895,1.5235599692
O,0,-1.183444558,-7.7588914778,-3.290399931
O,0,-1.9739257393,-9.2987355759,3.6719737518
N,0,2.5838621024,-1.1008617744,0.2698114583

N,0,2.4796364076,-3.6691841104,3.5581151803
N,0,3.2975402932,-1.7702803844,2.3410086351
C,0,1.6376610238,-3.1884148561,1.2981383991
C,0,1.6635583267,-3.9806826432,2.4933109034
C,0,0.1219166633,-4.8033862427,0.2811397726
C,0,0.0858424718,-5.5443217316,1.5101362413
C,0,0.8812512644,-3.6555081893,0.1918861214
C,0,2.4694400268,-2.0241509872,1.3190046099
C,0,0.8602752839,-5.1426550423,2.581102856
C,0,-0.6329261555,-6.5200961565,-1.3497026024
C,0,1.6189065371,-0.4878884793,-0.514504174
C,0,-0.8605202754,-7.4359041921,2.7480274104
C,0,3.269978822,-2.6119780932,3.413548504
C,0,-1.1283591537,-6.4046338103,-2.7802360835
C,0,-1.8781699168,-8.5238834452,2.4548040609
C,0,2.1223155639,0.3135728901,-1.6019447408
C,0,0.2400288251,-0.6123837306,-0.3050794129
C,0,1.2855399536,0.989911613,-2.4472040007
C,0,-0.6477907087,0.0234399647,-1.1688440741
C,0,-0.2013015954,0.982159123,-2.2103712204
C,0,-1.628463104,-7.8461019285,-4.6655759177
C,0,-2.9084197048,-10.4036485904,3.591553194
C,0,1.765327718,1.6658798138,-3.5959466066
C,0,2.1216724551,2.2391055348,-4.6085097244
H,0,3.4768213143,-0.6212118058,0.3085374177
H,0,0.8764524101,-3.1258266774,-0.7501447629
H,0,0.8926406565,-5.6898248151,3.5130646228
H,0,0.3842279615,-6.9246298485,-1.3220149436
H,0,-1.2899190704,-7.1552416138,-0.7571541575
H,0,0.108772408,-7.875445545,3.0064748362
H,0,-1.2004097514,-6.8030971721,3.5745726168
H,0,3.9513003272,-2.3772290274,4.2215489438
H,0,-0.4445826058,-5.7850302237,-3.3786503249
H,0,-2.1222513191,-5.9341504787,-2.7986831127
H,0,-1.5421523851,-9.1470619956,1.6132137276
H,0,-2.8479084557,-8.0790100002,2.1889368777
H,0,3.1932627325,0.3337618556,-1.7735082888
H,0,-0.139474749,-1.1774859488,0.534520373
H,0,-1.7140321322,-0.1224965242,-1.0718286727
H,0,-0.7479048932,0.9789687778,-3.155175023
H,0,-1.6210038175,-8.906212498,-4.9218170468
H,0,-0.9529650377,-7.3014969111,-5.3408391578
H,0,-2.6464448114,-7.4479108322,-4.7838163526
H,0,-2.8793076316,-10.8961064537,4.5639321207

H,0,-2.6159285957,-11.1180518815,2.8091605818
H,0,-3.9293201712,-10.0499882622,3.3896466232
H,0,2.4394757798,2.7474747826,-5.4879863288

$_{F}$IM[2]

Fe,0,-0.3999926108,3.6381720654,0.1588709459
N,0,-2.3552518546,4.1013368855,0.0617272328
C,0,-3.3085462585,3.8214095444,1.0289604417
C,0,-4.5938121881,4.3515892682,0.6215624923
C,0,-4.4172226109,4.94265051,-0.596767602
C,0,-3.02042651,4.7875124339,-0.9443580081
H,0,-5.5020378748,4.2689551318,1.2011290299
H,0,-5.1507668942,5.4428597492,-1.2125635846
C,0,-2.4339796817,5.2738807731,-2.102746395
H,0,-3.0686643443,5.8036168445,-2.8052961392
N,0,-0.1350740777,4.5185190939,-1.6283508606
C,0,-1.0870030266,5.1599619227,-2.411753166
C,0,-0.4584498349,5.7335372943,-3.5816400811
C,0,0.8756656743,5.4526334815,-3.4985449976
C,0,1.0780967787,4.6961013296,-2.2818364462
H,0,-0.9815750007,6.2831934753,-4.3509033221
H,0,1.6618647599,5.7236150091,-4.1883869366
C,0,2.303063146,4.2062773391,-1.857167542
H,0,3.1678790485,4.4230983185,-2.4754781318
N,0,1.5321753124,3.0697780985,0.1929853026
C,0,2.5098070867,3.4464188538,-0.7154166372
C,0,3.790039969,2.8965811724,-0.3184011865
C,0,3.5854138851,2.1911859678,0.8355632857
C,0,2.1785788573,2.3101524853,1.1578380322
H,0,4.7151229848,3.0477978531,-0.8564411285
H,0,4.2993178127,1.6307553912,1.4211831974
C,0,1.577647587,1.7523937183,2.2758273639
H,0,2.2041421442,1.1740117107,2.9458386814
N,0,-0.6991436501,2.59786799,1.8550589198
C,0,0.2389829122,1.8989973251,2.6037778072
C,0,-0.3788196684,1.3708103909,3.8013018588
C,0,-1.685709947,1.7671234176,3.7854383506
C,0,-1.8836641967,2.5360924507,2.574234186
H,0,0.1363276144,0.7867637944,4.5502384455
H,0,-2.4548307652,1.5696403457,4.518265596
C,0,-3.0900008376,3.1063823432,2.1965612868
H,0,-3.9355689976,2.9700287214,2.8627117625
O,0,-1.0742981587,1.7715352576,-0.9471540068

S,0,0.0961757483,5.5582109529,1.3244169285
H,0,-0.673904567,6.4300821113,0.5773970496
O,0,-0.671482675,-5.0979792899,-0.8526709334
O,0,-0.1394255846,-6.6446259239,1.3911971337
O,0,-1.5161945585,-7.6714142898,-3.2812662078
O,0,-0.6962951147,-9.4190227291,3.6629015481
N,0,2.4557881552,-0.8650212686,-0.378254138
N,0,3.2376931293,-3.4707433721,2.7871976531
N,0,3.6406601388,-1.4931552712,1.483970748
C,0,1.8996441912,-3.0169404508,0.7737692129
C,0,2.2345197415,-3.8229462411,1.9084943146
C,0,0.3031428257,-4.7148610829,0.0611480359
C,0,0.5901058741,-5.4813413529,1.2382778604
C,0,0.9470943745,-3.514651042,-0.1532379605
C,0,2.6365308137,-1.7918429407,0.6420248533
C,0,1.5495287845,-5.0421520243,2.1297744502
C,0,-0.5831365879,-6.4192009191,-1.498546833
C,0,1.256484447,-0.3413778774,-0.9141498202
C,0,0.069191797,-7.4618066916,2.5846531219
C,0,3.8993970764,-2.3540246333,2.5083616965
C,0,-1.5499768182,-6.3598661337,-2.6671229996
C,0,-0.9032976758,-8.622488461,2.4732924363
C,0,1.4128097558,0.3250809578,-2.1977466534
C,0,0.0313208033,-0.4444695902,-0.3199041063
C,0,0.3653523117,0.8972785928,-2.860738109
C,0,-1.120702845,0.2389651514,-0.8926604344
C,0,-0.9624879875,0.9671332788,-2.2048570344
C,0,-2.378023499,-7.794993263,-4.4376432168
C,0,-1.5441160664,-10.5914873697,3.7365003138
C,0,0.4875754153,1.4022744007,-4.1848859954
C,0,0.5536060975,1.8268739881,-5.3226269967
H,0,3.2458379949,-0.2275625667,-0.4327338106
H,0,0.6989519301,-2.966324399,-1.0508332886
H,0,1.8224057837,-5.601543638,3.0140435853
H,0,0.4396439273,-6.5952595827,-1.8493671761
H,0,-0.8656486581,-7.2020617334,-0.7952025986
H,0,1.1015478912,-7.8259389186,2.6205416642
H,0,-0.1354303732,-6.8735216192,3.4854249845
H,0,4.7186105044,-2.0805242078,3.1622632421
H,0,-1.2448498856,-5.5817787372,-3.3819708679
H,0,-2.5610160538,-6.1181300932,-2.3078628769
H,0,-0.699585633,-9.2078669468,1.5647713
H,0,-1.9364704665,-8.2501336931,2.4190447223
H,0,2.3893581572,0.2855058555,-2.667870613

H,0,-0.1052684904,-0.9955981242,0.6008796465
H,0,-2.1085488018,-0.0897154521,-0.593127088
H,0,-1.818742693,1.1068632048,-2.8546292763
H,0,-2.2588432055,-8.8170538858,-4.7994390353
H,0,-2.0891275596,-7.0902323129,-5.2307331796
H,0,-3.4311309414,-7.6203836538,-4.1738050039
H,0,-1.2884723316,-11.0923749415,4.6707463018
H,0,-1.3599575821,-11.2719115246,2.8930174923
H,0,-2.6081625155,-10.3157465639,3.7472785581
H,0,0.619101917,2.2108875611,-6.3131216474

$_F$TS[3]

Fe,0,-0.5946592246,3.7732273638,-0.3512940455
N,0,-2.5561755318,3.3191353054,-0.4424981974
C,0,-3.2639785747,2.5701912426,0.4813366943
C,0,-4.6518831368,2.4698825914,0.0723503211
C,0,-4.7746146269,3.1580166857,-1.1009004218
C,0,-3.4649069567,3.6907198041,-1.418717265
H,0,-5.4170332852,1.9414299709,0.6230297758
H,0,-5.6601769111,3.3062685037,-1.7022209372
C,0,-3.1764951393,4.4798126429,-2.5230816445
H,0,-3.9874529333,4.6985723929,-3.2100551398
N,0,-0.793620573,4.8572611951,-2.0338154378
C,0,-1.9328884307,5.0279350704,-2.8013417148
C,0,-1.6404606534,5.885251281,-3.9336082776
C,0,-0.3226095401,6.2294096352,-3.8425016085
C,0,0.2062910788,5.5792524647,-2.6577553669
H,0,-2.3604728476,6.1777433223,-4.6844610628
H,0,0.2528637562,6.860590329,-4.5047172422
C,0,1.5291148451,5.65872784,-2.2437178298
H,0,2.1960965832,6.2834438364,-2.8294571112
N,0,1.3877071814,4.1646687679,-0.285209516
C,0,2.0717040069,5.0057757797,-1.1469956769
C,0,3.4593077569,5.1045722396,-0.73894548
C,0,3.6069603729,4.3292676514,0.3753162012
C,0,2.3094975767,3.7524625188,0.6649370944
H,0,4.206154254,5.6998832998,-1.244703967
H,0,4.4969672602,4.1660245963,0.9662622864
C,0,2.0285046724,2.9469804405,1.759330301
H,0,2.8440093595,2.7156937754,2.4367635133
N,0,-0.3799688408,2.6521108837,1.3238550913
C,0,0.7700328091,2.4545142462,2.0762808341
C,0,0.4589274873,1.6734385469,3.2542182061

C,0,-0.8836792172,1.4158035169,3.2226002049
C,0,-1.4088993944,2.0337372931,2.0224449856
H,0,1.1789778295,1.3830628161,4.0060038568
H,0,-1.4778875638,0.8719605978,3.9432665784
C,0,-2.7382027059,1.9822856185,1.6249571647
H,0,-3.4275140105,1.4423262549,2.2663931111
O,0,-0.2454207924,2.1742505483,-1.6049179726
S,0,-1.0212096396,5.6280449568,0.9710983909
H,0,-2.0171925809,6.1883834631,0.1938284563
O,0,-0.7014458173,-5.0039723545,-0.5686936919
O,0,-0.5154206689,-6.7778946437,1.5781900381
O,0,-2.3998863177,-7.1303756623,-2.9792395055
O,0,-1.6652710804,-9.4612296574,3.7329305231
N,0,3.4053820131,-1.7313219344,-0.0106835098
N,0,3.6346946296,-4.689417649,2.9267294548
N,0,4.4757866734,-2.7975196626,1.7143980263
C,0,2.3700301115,-3.7693467335,1.0298184466
C,0,2.535549029,-4.7119912603,2.0970724397
C,0,0.3625021058,-4.9544513013,0.3155955311
C,0,0.4872233882,-5.8412992172,1.4382891998
C,0,1.2876514445,-3.9478116091,0.1298378271
C,0,3.3876755429,-2.7677840611,0.9362531396
C,0,1.566923829,-5.7255443243,2.2921162492
C,0,-1.1331140318,-6.279120319,-1.170407925
C,0,2.4015279206,-0.8594533316,-0.4205018832
C,0,-0.4744341679,-7.7017532513,2.7115349814
C,0,4.5549330939,-3.7669995822,2.67085505
C,0,-1.90396024,-5.8906563362,-2.4194980209
C,0,-1.726301544,-8.5547244023,2.6081977465
C,0,2.7245978197,0.0178462827,-1.5028704259
C,0,1.1324594355,-0.7934195793,0.1646554926
C,0,1.8409937663,0.9601840718,-1.9888732198
C,0,0.2087251744,0.1604798392,-0.2809435459
C,0,0.4866077635,1.1063707322,-1.3758328407
C,0,-3.1740275524,-6.9541158911,-4.1907617303
C,0,-2.792270214,-10.3689032696,3.8173194958
C,0,2.1820677683,1.8005427855,-3.076459963
C,0,2.4693277892,2.5142576636,-4.0165421169
H,0,4.353881389,-1.4152338724,-0.1824309168
H,0,1.1591580906,-3.3080467546,-0.7320620058
H,0,1.7210667592,-6.3935660278,3.128289383
H,0,-0.2609356756,-6.8906379294,-1.4229494623
H,0,-1.7668480953,-6.8255667536,-0.4736259104
H,0,0.422927598,-8.3271963544,2.6572299477

H,0,-0.4671129782,-7.142023398,3.6526375644
H,0,5.4488113853,-3.7621848386,3.2815158952
H,0,-1.2503277632,-5.3685802774,-3.1335312897
H,0,-2.7315244871,-5.215953035,-2.155904788
H,0,-1.7395109568,-9.1038685069,1.6554794029
H,0,-2.6247247877,-7.9224425218,2.654570875
H,0,3.7068794738,-0.0633612987,-1.9578617933
H,0,0.8597999622,-1.4411389059,0.9854603682
H,0,-0.7630031409,0.2615484553,0.1845397138
H,0,-0.0777790387,0.0405243803,-1.7897530113
H,0,-3.4836083319,-7.9523400675,-4.5024322545
H,0,-2.5729297301,-6.4917986978,-4.9867827299
H,0,-4.0635136516,-6.3337417722,-4.0105399726
H,0,-2.6175489702,-10.9869090866,4.6984235488
H,0,-2.8570764058,-11.0092550013,2.9264177162
H,0,-3.738068204,-9.8214341792,3.934403185
H,0,2.6988201753,3.152831813,-4.8366808804

sIM[3]

Fe,0,-0.6490242081,3.4335140136,-0.2172574657
N,0,-1.8140967939,1.7895816199,-0.2948982426
C,0,-2.2172809073,1.0192324532,0.7894742409
C,0,-3.1230339045,-0.018089521,0.3434105211
C,0,-3.2677792412,0.1268427837,-1.0083008041
C,0,-2.4623186823,1.2612517946,-1.4042725053
H,0,-3.5779472714,-0.7538971216,0.9907650129
H,0,-3.8724905626,-0.4610813016,-1.6838483472
C,0,-2.3960560313,1.7757474016,-2.6910839182
H,0,-2.952073771,1.2614834812,-3.4678843871
N,0,-0.9651556489,3.7145532368,-2.1817821067
C,0,-1.7102055932,2.9280403654,-3.0463116624
C,0,-1.7008426226,3.5095907028,-4.3729345562
C,0,-0.9545814882,4.6509262731,-4.3030852298
C,0,-0.4886162882,4.7734305908,-2.9361214946
H,0,-2.2077369263,3.0907859003,-5.2303192485
H,0,-0.7262899842,5.3516386751,-5.0933243915
C,0,0.3464287677,5.7826912161,-2.4793796701
H,0,0.6355473207,6.5490118091,-3.1910610203
N,0,0.6189617202,4.9948046265,-0.1589596473
C,0,0.8678637196,5.8751805695,-1.1978565079
C,0,1.7943760818,6.9014211658,-0.7610277595
C,0,2.1066194791,6.6327753975,0.5401733921
C,0,1.3649842786,5.4461279724,0.9172102821

H,0,2.1500026059,7.7107164372,-1.3823452394
H,0,2.7657306244,7.1794379688,1.1989993514
C,0,1.3734643319,4.8772761764,2.1820332687
H,0,1.9966319394,5.3418596407,2.9388122207
N,0,-0.2487009156,3.0765224272,1.7286456761
C,0,0.5998315751,3.7910195772,2.564347664
C,0,0.5180366227,3.2686375785,3.9113777914
C,0,-0.4009077826,2.2568852506,3.8951218936
C,0,-0.8813984528,2.138028981,2.5342904382
H,0,1.0814561752,3.6479432683,4.7517046305
H,0,-0.7341390206,1.6421276639,4.7190005666
C,0,-1.796239706,1.1885036126,2.1019104769
H,0,-2.2035765628,0.5126537432,2.846701651
O,0,1.0765492567,2.2883438582,-0.8653306088
S,0,-2.4079638186,4.7982054565,0.3435733974
H,0,-3.3190553988,4.3184464913,-0.5790186925
O,0,-0.7840280335,-4.1349208899,-0.3507065791
O,0,-0.7326258667,-6.5328727456,1.0266944136
O,0,-3.4227899192,-4.8949140872,-2.7371494357
O,0,-2.2373067226,-9.4672604138,2.540220006
N,0,4.1861112361,-2.2463701587,-0.0019055926
N,0,3.9954566806,-5.9626605543,1.8828598074
N,0,5.1398878189,-4.0104934856,1.0825510773
C,0,2.7640063442,-4.2133519166,0.6620545133
C,0,2.821614694,-5.4651057768,1.3566807495
C,0,0.4070971265,-4.6174201692,0.1807146273
C,0,0.4580268429,-5.8390736995,0.927807721
C,0,1.5372584855,-3.8388646433,0.0524813691
C,0,4.000982225,-3.4837003489,0.5998536687
C,0,1.650682699,-6.2512437992,1.4889424389
C,0,-1.4731546763,-4.9194072141,-1.3899734941
C,0,3.3205529883,-1.1289573512,-0.1148568449
C,0,-0.7717519889,-7.7665988674,1.8088827468
C,0,5.0840008494,-5.2272377303,1.6892160905
C,0,-2.7550165751,-4.1613951107,-1.6813150541
C,0,-2.2101215029,-8.250023139,1.7592992162
C,0,3.6122205896,-0.2337528483,-1.2203800788
C,0,2.3082682192,-0.8382749789,0.74118692
C,0,2.9063454501,0.9126493831,-1.4805176877
C,0,1.4236590799,0.3472342822,0.5324296547
C,0,1.757703823,1.2607048093,-0.622199278
C,0,-4.6779477926,-4.3058057484,-3.1512281396
C,0,-3.5435198943,-10.0903820114,2.6080838254
C,0,3.2368534374,1.7665415636,-2.5707874652

C,0,3.5215640347,2.4929656712,-3.5014393416
H,0,5.1765377604,-2.0521171805,-0.1097467306
H,0,1.4497846421,-2.932349507,-0.5279098002
H,0,1.7471179043,-7.1775309418,2.0385666035
H,0,-0.8443620169,-4.9735786639,-2.2855677879
H,0,-1.6870259793,-5.9251682522,-1.0270191817
H,0,-0.0992404844,-8.5140902784,1.3744732254
H,0,-0.4680146584,-7.5682965903,2.8422323043
H,0,6.0247563682,-5.6162867603,2.0594311981
H,0,-2.5284013252,-3.1319447783,-1.9950823693
H,0,-3.3802214273,-4.1138066409,-0.7777952012
H,0,-2.5156460157,-8.4394573891,0.7198688802
H,0,-2.8828054473,-7.4904536591,2.182985911
H,0,4.4345931412,-0.5020795651,-1.8772102537
H,0,2.0989014654,-1.4710648019,1.5959355273
H,0,1.3948747176,0.9650929474,1.4428621743
H,0,0.374374555,0.0314081597,0.4103054
H,0,-5.0702353414,-4.9461171112,-3.942324332
H,0,-4.5337865828,-3.2882991262,-3.5431787604
H,0,-5.3976163977,-4.267662265,-2.3205959265
H,0,-3.4210424641,-10.9854854146,3.2186776593
H,0,-3.9037957368,-10.3742540641,1.6090955228
H,0,-4.2808478857,-9.4239536319,3.077765721
H,0,3.7601716408,3.137633257,-4.314164608

FTS[4]

Fe,0,-0.669953429,3.7777721186,-0.3509275968
N,0,-2.6433861858,3.413598243,-0.5288356633
C,0,-3.430316431,2.728653658,0.3826597174
C,0,-4.8014256725,2.6852524228,-0.0850028192
C,0,-4.8377851434,3.3429817755,-1.2813931227
C,0,-3.4903486237,3.7978031915,-1.555392883
H,0,-5.615387728,2.2114456274,0.4448053291
H,0,-5.687292439,3.5158068261,-1.9264054659
C,0,-3.1115659367,4.5276093349,-2.6733583156
H,0,-3.8793421061,4.7664540608,-3.4019085577
N,0,-0.7312169351,4.7795305535,-2.0938502207
C,0,-1.8275430974,4.9876549,-2.9183903959
C,0,-1.4374092628,5.7783568979,-4.0680635722
C,0,-0.1061893037,6.0486209104,-3.932702723
C,0,0.3334411727,5.4197568157,-2.7019952985
H,0,-2.1070234501,6.0813169689,-4.8601579448
H,0,0.5325129452,6.6186940631,-4.5922966394

C,0,1.6383411982,5.4460211291,-2.2307690479
H,0,2.3648622004,6.0074969691,-2.8093387835
N,0,1.3214933241,4.0537477547,-0.217796982
C,0,2.092069683,4.8173330026,-1.0819755881
C,0,3.4590103365,4.8733525151,-0.6061119609
C,0,3.510289609,4.1589339034,0.5561142895
C,0,2.1746142283,3.6599968115,0.8064033315
H,0,4.2592790148,5.4025164713,-1.1033607077
H,0,4.3594665085,3.9884710071,1.2022420997
C,0,1.7993238792,2.9381289626,1.9279201934
H,0,2.5660739813,2.7093101042,2.6605548633
N,0,-0.5900150523,2.7211244054,1.367464013
C,0,0.5068191991,2.5096742379,2.19256223
C,0,0.1026628633,1.7807317386,3.3746538038
C,0,-1.2427738058,1.5626672369,3.2710228638
C,0,-1.6765302128,2.1580817604,2.0242971554
H,0,0.7689088894,1.4906577937,4.1744046755
H,0,-1.8953350144,1.0590127544,3.9698411133
C,0,-2.9853123716,2.1512666079,1.5633792601
H,0,-3.7266404545,1.6579281356,2.1838571403
O,0,-0.3751898059,2.0875133944,-1.5426707839
S,0,-1.0652052651,5.716573184,0.8596238536
H,0,-2.0217357145,6.2616655199,0.0240572664
O,0,-0.6895602813,-4.9426707763,-0.4636523552
O,0,-0.3984636139,-6.816504319,1.5776832813
O,0,-2.5215244568,-6.9186650632,-2.9052440465
O,0,-1.4486201871,-9.5932412802,3.6641330272
N,0,3.5211834296,-1.7614568347,-0.0157380886
N,0,3.8558381987,-4.8482590024,2.7720148636
N,0,4.6635211059,-2.9215697685,1.5916559751
C,0,2.4982428865,-3.8221226694,0.9950332872
C,0,2.7093240643,-4.8140570683,2.0082656844
C,0,0.4311594969,-4.9450908524,0.3526916178
C,0,0.6076847183,-5.884457272,1.4228237765
C,0,1.3600502668,-3.9443311172,0.1559290743
C,0,3.5305736443,-2.8345556404,0.8802018486
C,0,1.7367828884,-5.8212849749,2.2160910557
C,0,-1.1212204513,-6.1755926115,-1.1462714889
C,0,2.499193311,-0.8951052678,-0.4058524912
C,0,-0.3015023682,-7.7928221844,2.6616536704
C,0,4.7772481462,-3.9316082244,2.4991284887
C,0,-2.0381114411,-5.7132172549,-2.264451021
C,0,-1.5624742143,-8.6351904579,2.5868130856
C,0,2.758207166,-0.035253843,-1.4951626007

C,0,1.2598269459,-0.8023087274,0.275712641
C,0,1.8228142821,0.905979239,-1.9242716407
C,0,0.3138946782,0.1143105075,-0.1360014975
C,0,0.5229648921,0.9538610231,-1.2797664094
C,0,-3.4163540669,-6.6690451215,-4.0159940197
C,0,-2.5725917085,-10.5028052882,3.7618148871
C,0,2.1010294062,1.7455271477,-3.0341449362
C,0,2.3420865115,2.4437256161,-3.9989952748
H,0,4.4647159897,-1.4395518109,-0.2027793471
H,0,1.1875877989,-3.2628025576,-0.6651803315
H,0,1.9299332639,-6.528418413,3.0109568058
H,0,-0.2516961155,-6.7053675532,-1.549620413
H,0,-1.6491843794,-6.825373222,-0.4496725315
H,0,0.5874278216,-8.4187325886,2.5292852626
H,0,-0.2390057308,-7.2788899429,3.6266042386
H,0,5.7058109486,-3.9698006054,3.0548454039
H,0,-1.4909118841,-5.079345576,-2.9773881051
H,0,-2.8698160676,-5.1244949925,-1.8506426454
H,0,-1.6295835817,-9.1401870779,1.6121617897
H,0,-2.4531869888,-8.002307507,2.7096097466
H,0,3.7094800407,-0.1044809761,-2.0122430001
H,0,1.0693815814,-1.4189893972,1.1436942537
H,0,-0.6235968455,0.2144596478,0.3953462855
H,0,-0.3734837413,0.7810574019,-2.0711212185
H,0,-3.7022065664,-7.6477832447,-4.4032265457
H,0,-2.9196106,-6.0923171753,-4.8094197433
H,0,-4.3158949099,-6.1262326824,-3.6920061947
H,0,-2.3570318114,-11.1609547397,4.6040129861
H,0,-2.6829336041,-11.1016124618,2.846729466
H,0,-3.5101904737,-9.9609628694,3.9499813535
H,0,2.5296120141,3.0697036624,-4.8389340366

$_F$P[4]

Fe,0,-0.8420424669,3.7686534379,-0.2148640337
N,0,-2.6978215531,3.2025267395,-0.7458342031
C,0,-3.6089716951,2.5433692574,0.0666834555
C,0,-4.8568983816,2.3549741666,-0.6434145413
C,0,-4.7002237649,2.9011837882,-1.884813133
C,0,-3.3553287667,3.4322499334,-1.9481070885
H,0,-5.7278406224,1.8663015715,-0.2311950908
H,0,-5.4172125883,2.9501150922,-2.6915318858
C,0,-2.8180662793,4.0892815666,-3.0453646497
H,0,-3.451931373,4.2161405714,-3.9163906185

N,0,-0.5905636787,4.522493419,-2.0785493511
C,0,-1.5354482414,4.6145943089,-3.0999809655
C,0,-0.9853185885,5.3536511198,-4.2117068895
C,0,0.2837272323,5.7259747644,-3.8607983356
C,0,0.5290971275,5.2160371351,-2.5314226584
H,0,-1.5138520834,5.5631826588,-5.130529666
H,0,0.9950599376,6.3002379858,-4.4369025788
C,0,1.7117195305,5.383673928,-1.8269293377
H,0,2.5069731323,5.9366856196,-2.3155145019
N,0,1.0726334943,4.1919805181,0.2403899994
C,0,1.9623328785,4.9068914077,-0.549024182
C,0,3.2120650095,5.0908550319,0.15973722
C,0,3.0736869384,4.4954954581,1.3803051173
C,0,1.7371898236,3.9427166443,1.4332706028
H,0,4.0704568051,5.612383441,-0.2382231178
H,0,3.795399806,4.4327567927,2.1817075627
C,0,1.1958310248,3.2886034797,2.5289661257
H,0,1.8228783403,3.1715613999,3.4062430539
N,0,-1.0275032704,2.8503220363,1.5609203117
C,0,-0.096554542,2.7911800155,2.5902617198
C,0,-0.6751445801,2.1327741943,3.7413511803
C,0,-1.9599617667,1.8056143117,3.4121713194
C,0,-2.1820530885,2.2603543396,2.055568713
H,0,-0.1561380899,1.9564047935,4.6722908796
H,0,-2.7011650352,1.3077341763,4.0203835742
C,0,-3.3740082081,2.1117780743,1.362932535
H,0,-4.1901913015,1.6160842954,1.8778647505
O,0,-0.1261749186,1.8841943428,-1.2706639512
S,0,-1.5689781596,5.758957707,0.6565799233
H,0,-2.4426685095,6.0979000428,-0.3594468072
O,0,-0.699369456,-4.5946452124,-0.2276696312
O,0,-0.2745848066,-6.6825478966,1.5406609175
O,0,-3.1079441405,-6.1351316106,-2.4790497671
O,0,-1.2950377316,-9.5086113355,3.5749402406
N,0,3.897211621,-1.9162368395,-0.2962375882
N,0,4.32399197,-5.2886190177,2.115921985
N,0,5.1453790525,-3.3395294545,0.9780476413
C,0,2.8190147053,-3.9549302938,0.6927212773
C,0,3.0786010068,-5.0586909178,1.5676532944
C,0,0.5507309135,-4.7843634875,0.3550459265
C,0,0.7951328365,-5.8431731843,1.287457202
C,0,1.5435896552,-3.8699185294,0.0738598829
C,0,3.9281495633,-3.0651174982,0.4723151815
C,0,2.0425805669,-5.976947736,1.865336046

C,0,-1.2508504821,-5.6588019725,-1.0829220734
C,0,2.8374706978,-1.002139256,-0.5457904977
C,0,-0.1141671976,-7.7578602881,2.5156535221
C,0,5.2842672871,-4.4413718473,1.7628098371
C,0,-2.5386378247,-5.0913217757,-1.6503513087
C,0,-1.4644832773,-8.4457879809,2.607775432
C,0,2.8198650219,-0.319360213,-1.7668900727
C,0,1.8600463891,-0.7033950202,0.4233508392
C,0,1.8487596257,0.6654667493,-2.0320437626
C,0,0.8717569013,0.2437058536,0.1634537259
C,0,0.8710067029,0.933966912,-1.0481186454
C,0,-4.3450726168,-5.7529314442,-3.1251516984
C,0,-2.4920277022,-10.2929158865,3.7981204515
C,0,1.8164463433,1.3746050955,-3.2707081119
C,0,1.7399041771,2.0035954541,-4.3089842649
H,0,4.8352661636,-1.5762102299,-0.4763922894
H,0,1.3116346814,-3.0892299697,-0.63619959
H,0,2.2848429677,-6.778351523,2.5498454672
H,0,-0.5428801725,-5.892116875,-1.8861457381
H,0,-1.4447682511,-6.5554325092,-0.4935226284
H,0,0.6549121953,-8.4631756364,2.1826491392
H,0,0.175064063,-7.3461644496,3.4884869879
H,0,6.280612116,-4.6271110406,2.1460748207
H,0,-2.3333167839,-4.1870682472,-2.2415167808
H,0,-3.2229813168,-4.8190697874,-0.8336940004
H,0,-1.758205641,-8.8469378927,1.6267610759
H,0,-2.2351316818,-7.7321139615,2.9328285154
H,0,3.5591658635,-0.5502562934,-2.5254686308
H,0,1.8748102022,-1.2129169557,1.3791638517
H,0,0.1117707101,0.4716623029,0.8981148964
H,0,-0.0751328055,2.2834091411,-2.1682466663
H,0,-4.6621363677,-6.6152212535,-3.7132474044
H,0,-4.20044639,-4.8894038647,-3.7904951324
H,0,-5.1235456821,-5.5060442227,-2.3885977625
H,0,-2.2267660698,-11.0471810907,4.5397054727
H,0,-2.8245638713,-10.7878002036,2.8745060963
H,0,-3.3120483674,-9.6711653097,4.1848500408
H,0,1.6813151094,2.5610069387,-5.2140401782

$_{H}$IM[1]

Fe,0,0.5496654201,4.187715794,0.1128860629
N,0,-1.3409251134,4.3838505076,-0.5398876658
C,0,-2.4978843632,4.2573990783,0.2057070604

C,0,-3.6569622112,4.378441834,-0.6502426474
C,0,-3.1949239713,4.5792190242,-1.9199266882
C,0,-1.7508383121,4.5840436336,-1.8470676684
H,0,-4.6801458348,4.3178300781,-0.3099908483
H,0,-3.7641755825,4.7161231792,-2.8274549936
C,0,-0.9084877013,4.7775558036,-2.9317342505
H,0,-1.3710507384,4.9209948508,-3.9024437128
N,0,1.2351517159,4.6469514131,-1.7264412989
C,0,0.4762304096,4.8035105703,-2.8760808732
C,0,1.3414687997,5.0012990152,-4.0170098634
C,0,2.6267367878,4.9567760562,-3.556081157
C,0,2.5563021668,4.7326281526,-2.1296599637
H,0,0.9967692902,5.1517049405,-5.0294423369
H,0,3.544327543,5.0634663187,-4.1156475684
C,0,3.6607117591,4.6291908108,-1.2920037345
H,0,4.6410748866,4.7140430658,-1.7485051708
N,0,2.4430483007,4.2806372068,0.8212800646
C,0,3.6108689589,4.4223041032,0.0742092311
C,0,4.7641277442,4.298022119,0.9369435297
C,0,4.300435362,4.0582177523,2.1975507682
C,0,2.8569953982,4.0382828357,2.1271424909
H,0,5.7883502959,4.3762286093,0.6034351771
H,0,4.86885636,3.8991556451,3.1016843892
C,0,2.017080535,3.8026763904,3.2002037942
H,0,2.4781367522,3.6047318015,4.1610558272
N,0,-0.1271433255,4.0048940626,2.004606105
C,0,0.6316870145,3.8035638222,3.1459943012
C,0,-0.2338814435,3.6043589336,4.2874278221
C,0,-1.5189499389,3.6873420875,3.8362270151
C,0,-1.4533020641,3.9289694594,2.4118429496
H,0,0.1138729628,3.4247462335,5.2937059549
H,0,-2.4353387009,3.5906194694,4.3993398416
C,0,-2.5512666356,4.0512662979,1.5788159878
H,0,-3.5332956451,3.9721423188,2.0327063905
O,0,0.6205034448,2.5453999036,-0.1347377504
S,0,0.671064914,6.647631489,0.7701571346
H,0,1.7034234185,7.0314512751,-0.0653704719
O,0,0.3668883766,-4.5487221123,-1.5280299981
O,0,-1.3155878731,-6.3660376662,-2.7633206383
O,0,3.645754289,-5.7279232814,-2.576288317
O,0,-2.3974740484,-8.4715426206,-5.5132767448
N,0,-2.1310447798,-3.2361379004,2.9894762787
N,0,-4.38652249,-6.0989850088,0.969152494
N,0,-3.9099830902,-4.6518192091,2.8273098207

C,0,-2.3530804035,-4.703188624,0.9667190892
C,0,-3.2243303983,-5.6657064003,0.3616527629
C,0,-0.8095752678,-4.9605032979,-0.9045752671
C,0,-1.7100053442,-5.8788497268,-1.5284380936
C,0,-1.1326006869,-4.3933265207,0.3102348269
C,0,-2.7893853618,-4.1828553587,2.239210712
C,0,-2.8865933553,-6.2268267944,-0.8939091112
C,0,1.3960208699,-5.5538290205,-1.834732992
C,0,-1.1889335508,-2.2351896037,2.5928864897
C,0,-2.204490718,-7.2725487478,-3.4818022725
C,0,-4.641960132,-5.5868083754,2.1681157308
C,0,2.6313909459,-4.7574275549,-2.2127811498
C,0,-1.512000622,-7.5771351322,-4.7981366234
C,0,-0.0020155198,-2.0878647856,3.316038453
C,0,-1.4662999618,-1.3448897492,1.5403674289
C,0,0.9205945882,-1.0675371256,3.0103814696
C,0,-0.555456404,-0.3462963568,1.2044664307
C,0,0.637064912,-0.1915018706,1.9327062201
C,0,4.9150041317,-5.1322061689,-2.9316708837
C,0,-1.893785972,-8.8770860957,-6.8086018069
C,0,2.1183983188,-0.9300726289,3.7744283103
C,0,3.1261007346,-0.8419041118,4.4483798101
H,0,-2.55483584,-3.1358552028,3.9052138609
H,0,-0.4205807888,-3.7038883296,0.7389096219
H,0,-3.5881587599,-6.9346426784,-1.3139196241
H,0,1.5891081677,-6.1730516763,-0.9514271591
H,0,1.0713404954,-6.1886562988,-2.660124062
H,0,-2.3606453812,-8.1920464107,-2.9069529972
H,0,-3.1735565942,-6.7933158777,-3.6582409489
H,0,-5.5322448319,-5.9356173274,2.6788791746
H,0,2.9640197538,-4.1389330269,-1.3668398956
H,0,2.4049379507,-4.0902949439,-3.057235123
H,0,-0.5359287735,-8.0502124591,-4.6164204335
H,0,-1.3447177492,-6.6491810781,-5.3637299054
H,0,0.2201560134,-2.7735680972,4.1261304707
H,0,-2.3930427691,-1.4433349485,0.9853976668
H,0,-0.7626127007,0.3346614815,0.386117165
H,0,5.5835700621,-5.9587675746,-3.1768261417
H,0,5.3369312063,-4.5562084185,-2.09520661
H,0,4.8180250018,-4.4691787807,-3.8039781585
H,0,-2.6475400956,-9.5406243362,-7.2340872946
H,0,-0.9402667528,-9.41671333,-6.7169022076
H,0,-1.7507800217,-8.011852116,-7.4718196635
H,0,4.016406055,-0.7676386682,5.0258174336

O,0,1.5527645826,0.7820605909,1.6413022413
H,0,1.2518489247,1.399010414,0.9119539275

$_H$TS[1]

Fe,0,-0.8967431011,-1.0622656492,-0.4029896356
N,0,-2.4874831599,-1.4364513855,-1.5914501322
C,0,-3.7678977309,-1.7746213523,-1.1931618457
C,0,-4.601306199,-2.0056055837,-2.354285538
C,0,-3.8186713274,-1.8018236195,-3.4538419021
C,0,-2.5004165648,-1.4421617302,-2.9744649858
H,0,-5.6450099962,-2.2810394726,-2.3146431079
H,0,-4.0922551842,-1.8768054632,-4.4960647681
C,0,-1.4242935051,-1.1320167529,-3.7908125998
H,0,-1.5812737144,-1.1816979897,-4.8630376394
N,0,0.2222806414,-0.6331620329,-2.025054986
C,0,-0.1643530675,-0.7558554592,-3.3492355885
C,0,0.9497528817,-0.445404257,-4.2175891674
C,0,2.0132544056,-0.1462113125,-3.4140487917
C,0,1.5595344489,-0.2708889779,-2.0468206874
H,0,0.9081846938,-0.4620703267,-5.2968184361
H,0,3.0162975073,0.1307935336,-3.7037787758
C,0,2.3555602683,-0.0625989723,-0.9289494981
H,0,3.3893960785,0.2189358749,-1.0978332833
N,0,0.6556626345,-0.5433093754,0.7841651394
C,0,1.933878276,-0.1894445067,0.3845946374
C,0,2.7669488283,0.0353918894,1.5469742555
C,0,1.9898275128,-0.192774328,2.6452526358
C,0,0.6730934734,-0.5555491813,2.1656778251
H,0,3.8066125111,0.3256090898,1.5083852197
H,0,2.264683357,-0.1289516568,3.687651691
C,0,-0.4042918035,-0.8669679638,2.9810687828
H,0,-0.2392509563,-0.8464486648,4.0526065195
N,0,-2.0743490602,-1.2874352444,1.2185355561
C,0,-1.6800542121,-1.1904184176,2.5405110889
C,0,-2.7992704886,-1.4809554002,3.409724571
C,0,-3.8702893079,-1.7528801156,2.6068583816
C,0,-3.4146656328,-1.6379439272,1.2388907317
H,0,-2.754721454,-1.4718468666,4.4887782357
H,0,-4.8781145742,-2.0110501311,2.8971525231
C,0,-4.2030137848,-1.8644517483,0.1210237979
H,0,-5.2407601894,-2.1327654735,0.2875104956
O,0,-0.4132865676,-2.7064147053,-0.3627967506
S,0,-1.595479358,1.26877918,-0.3288495362

H,0,-0.528273921,1.8137294663,-1.0164263603
O,0,-1.0299788708,-9.3500849092,-2.0757673425
O,0,-3.0590611025,-10.9440068858,-3.1202914737
O,0,1.8061168367,-10.4647861966,-4.060099919
O,0,-4.8327370899,-12.6811536558,-5.7663896519
N,0,-2.4170943258,-8.50889941,2.9784554336
N,0,-5.1699293404,-11.1042455659,1.2298130733
N,0,-4.21325319,-9.9117050285,3.0802745055
C,0,-3.1490081115,-9.7331025014,0.9099591118
C,0,-4.1733270611,-10.6068538315,0.4173188413
C,0,-2.0665861259,-9.805366786,-1.2746365341
C,0,-3.1366286261,-10.612786992,-1.7810176267
C,0,-2.0856501731,-9.3817237912,0.0385215021
C,0,-3.267293039,-9.3657674966,2.2943719505
C,0,-4.1550572572,-11.0136980134,-0.9384828143
C,0,-0.2566089255,-10.2898269225,-2.9049741542
C,0,-1.7151228447,-7.361820564,2.5627459732
C,0,-4.1245012061,-11.7462783399,-3.7161918002
C,0,-5.1122208691,-10.758949457,2.5120979484
C,0,1.0630806243,-9.5896476173,-3.1756133737
C,0,-3.7716993379,-11.8870642963,-5.1862292895
C,0,-0.6197866288,-6.9414671557,3.3375885756
C,0,-2.1207811825,-6.5726833965,1.4574130669
C,0,0.0893836534,-5.7734004008,3.0316329881
C,0,-1.4312336948,-5.4201951329,1.1311179316
C,0,-0.3151574749,-4.9821581882,1.9003166826
C,0,3.1137072168,-9.9556185828,-4.4156857549
C,0,-4.6714304656,-12.9251647167,-7.1852624681
C,0,1.1975663152,-5.3705805353,3.8278785937
C,0,2.1410863808,-5.0473839037,4.523462264
H,0,-2.5047728201,-8.6385894092,3.9807704257
H,0,-1.256692371,-8.774773779,0.3735522099
H,0,-4.9647850253,-11.6509435361,-1.2663990625
H,0,-0.0970303198,-11.2282598368,-2.3636392356
H,0,-0.7885539954,-10.495920824,-3.8331950056
H,0,-4.1738454652,-12.7295842096,-3.2360735991
H,0,-5.0889230786,-11.2408235098,-3.5986550667
H,0,-5.8585057827,-11.1758838555,3.1773730935
H,0,1.6111686074,-9.4196310694,-2.2376182006
H,0,0.8810230084,-8.6131771675,-3.6473251261
H,0,-2.7972782883,-12.3837991882,-5.3008350498
H,0,-3.7118448717,-10.89682935,-5.660470756
H,0,-0.3031316317,-7.541004073,4.1848198939
H,0,-2.9833286426,-6.8671274687,0.8723114884

H,0,-1.7332013072,-4.8148359316,0.2841875844
H,0,3.5621137453,-10.6992887663,-5.0758533204
H,0,3.7483907642,-9.8245181823,-3.5274402625
H,0,3.0397020061,-8.993976456,-4.9436253013
H,0,-5.5278643295,-13.5281484301,-7.4886734009
H,0,-3.7429424856,-13.4754553109,-7.3936342404
H,0,-4.6645192393,-11.9848682366,-7.7546498344
H,0,2.973506187,-4.7574822545,5.1195167188
O,0,0.3442186043,-3.8687854719,1.6145532513
H,0,0.0043219891,-3.2912524001,0.6394434849

$_{H}$IM[2]

Fe,0,0.4289779241,3.9051472159,-0.0761829755
N,0,-0.9265363942,3.4068870811,-1.4797539641
C,0,-2.2221626895,2.9700324569,-1.2619401562
C,0,-2.8625311151,2.6949064751,-2.5308516881
C,0,-1.9545693315,2.9802030287,-3.5095716234
C,0,-0.74612938,3.4308669447,-2.8516606619
H,0,-3.8771682539,2.3400091456,-2.6389736485
H,0,-2.0753439457,2.9043161844,-4.5804396305
C,0,0.4016741826,3.837412597,-3.5108501251
H,0,0.3992900477,3.8004600278,-4.5946193777
N,0,1.747403694,4.3989404891,-1.5194304229
C,0,1.5579288696,4.2778558834,-2.8868691978
C,0,2.7715122194,4.6501401467,-3.5795980601
C,0,3.6969880968,4.9737024634,-2.6279963073
C,0,3.0616546315,4.8065482944,-1.3399944913
H,0,2.8865526273,4.6479437035,-4.6537699332
H,0,4.7204581928,5.2900618003,-2.7675810627
C,0,3.6891101839,5.0110441359,-0.121480426
H,0,4.7221953721,5.3400890982,-0.1379659275
N,0,1.7896668698,4.4129076812,1.3326830122
C,0,3.0919142198,4.8338850023,1.1159717502
C,0,3.7310872969,5.1065141645,2.3848499688
C,0,2.8079173165,4.8648925149,3.361101519
C,0,1.5921094502,4.4384966256,2.7039275586
H,0,4.7514578218,5.4437412395,2.4943087449
H,0,2.9210714363,4.962669507,4.430822875
C,0,0.4111785475,4.129348008,3.3575009734
H,0,0.4048904885,4.1945034404,4.439772744
N,0,-0.9453064323,3.5948899335,1.3651102068
C,0,-0.7621293904,3.7390559063,2.7321309138
C,0,-1.981787147,3.3846928078,3.4231683321

C,0,-2.8926170272,3.0181194417,2.472864691
C,0,-2.2435552862,3.1455008597,1.1869428407
H,0,-2.1065157486,3.4143298431,4.4958182
H,0,-3.9121088668,2.6891421654,2.6125880501
C,0,-2.8386394988,2.8453599643,-0.0284649517
H,0,-3.866486336,2.4999761416,-0.0123268714
O,0,1.0062400733,2.210212709,-0.0232295196
S,0,-0.3691015937,6.1302622945,-0.0551884672
H,0,0.5865539568,6.6918466591,-0.8796902569
O,0,0.4647755463,-4.4270464424,-1.58454696
O,0,-1.7577263844,-5.7995495851,-2.5605771421
O,0,3.0528547079,-5.7490514571,-3.7684941553
O,0,-3.8017252346,-7.3285995037,-5.1393347372
N,0,-0.6063282788,-3.527200119,3.518376771
N,0,-3.6833803395,-5.81521296,1.8762892273
N,0,-2.5347422052,-4.7454360646,3.6906262427
C,0,-1.5574846377,-4.6419492433,1.4775137374
C,0,-2.6827064834,-5.4065385004,1.0223191909
C,0,-0.5781943446,-4.7898965161,-0.7505811376
C,0,-1.7451913259,-5.4822137194,-1.217909221
C,0,-0.5012531419,-4.3838632461,0.5659537487
C,0,-1.5830771525,-4.2864669323,2.8653073208
C,0,-2.7618420454,-5.7944269745,-0.3364004704
C,0,1.0829704377,-5.4016142028,-2.5008301506
C,0,0.1051979166,-2.3995892981,3.1161072668
C,0,-2.9222055761,-6.484419876,-3.1195170298
C,0,-3.5389368153,-5.4960418624,3.1576438529
C,0,2.4567896142,-4.8362828617,-2.8143223337
C,0,-2.6420475386,-6.6512840018,-4.6024250451
C,0,1.2082940377,-2.0066993201,3.9101636225
C,0,-0.2791985956,-1.5968324674,2.0017528206
C,0,1.9507931642,-0.8679687858,3.6140000589
C,0,0.4352201443,-0.4679881295,1.6831207745
C,0,1.5820422457,-0.0496920923,2.457371516
C,0,4.3843127544,-5.3634652853,-4.1874346798
C,0,-3.7241291096,-7.5830545501,-6.5638533318
C,0,3.065333958,-0.4943177916,4.4100408668
C,0,4.018414882,-0.1842207944,5.098616432
H,0,-0.5944109893,-3.7295063287,4.5125256441
H,0,0.3970411182,-3.8666839698,0.8724057987
H,0,-3.6426805575,-6.3447829181,-0.6368976501
H,0,1.1643739245,-6.3789079946,-2.013891651
H,0,0.4833513291,-5.4983779469,-3.4048608282
H,0,-3.0538520026,-7.4606489479,-2.6407226207

H,0,-3.8235816884,-5.882521935,-2.9633781903
H,0,-4.2912169662,-5.8476310534,3.8524841779
H,0,3.0655401823,-4.7671050122,-1.9011153793
H,0,2.3607382208,-3.8266313754,-3.2389506878
H,0,-1.7294361061,-7.2453053135,-4.7560777094
H,0,-2.4990565217,-5.6698716516,-5.077070427
H,0,1.493352459,-2.6197334374,4.7594456312
H,0,-1.1517034182,-1.8671473885,1.4208547124
H,0,0.1511105816,0.1668647231,0.8497016001
H,0,4.7136503983,-6.1246022716,-4.8959222269
H,0,5.0788395478,-5.330831722,-3.3357380817
H,0,4.3803855382,-4.3808045426,-4.6803190208
H,0,-4.6511295683,-8.0908861217,-6.8317434148
H,0,-2.8682955615,-8.2278342967,-6.8087120875
H,0,-3.6408378873,-6.6466256596,-7.1332617161
H,0,4.856695492,0.0994970542,5.6900446528
O,0,2.2495712762,1.0072982749,2.1511782492
H,0,1.5941469169,1.9347163369,0.7343255361
